# Oral rehydration for severe malnutrition in children with moderate and severe dehydration (GASTROSAM): a phase 2, open-label, superiority randomised controlled trial

**DOI:** 10.1016/S2352-4642(25)00371-2

**Published:** 2026-03-10

**Authors:** Kathryn Maitland, Hadiza Sainna, Roisin Connon, San Maurice Ouattara, Temmy Sunyoto, Abdullahi Chara, Oluwakemi F Ogundipe, Amadou Saidou Maguina, Mainga Hamaluba, Peter Olupot-Olupot, Florence Alaroker, William Okiror, Denis Aromut, Eric Mwajombo, Emmanuel Oguda, Christabel Mogaka, Céline Langendorf, Juan Emmanuel Dewez, Iza Ciglenecki, Diana M Gibb, Roberta Petrucci, Matthew E Coldiron, Elizabeth C George

**Affiliations:** Institute of Global Health and Innovation, Department of Surgery and Cancer, Division of Medicine, Imperial College London, London, UK; Kenya Medical Research Institute (KEMRI)–Wellcome Trust Research Programme, Kilifi, Kenya; Médecins Sans Frontières, Maiduguri, Nigeria; Medical Research Council, Clinical Trials Unit at University College London, London, UK; Epicentre, Magaria, Niger; Médecins Sans Frontières, Brussels, Belgium; Médecins Sans Frontières, Maiduguri, Nigeria; Médecins Sans Frontières, Brussels, Belgium; Médecins Sans Frontières, Magaria, Niger; Kenya Medical Research Institute (KEMRI)–Wellcome Trust Research Programme, Kilifi, Kenya; Busitema University Faculty of Health Sciences, Mbale Regional Referral Hospital Mbale, Mbale, Uganda; Soroti Regional Referral Hospital, Soroti, Uganda; Busitema University Faculty of Health Sciences, Mbale Regional Referral Hospital Mbale, Mbale, Uganda; Soroti Regional Referral Hospital, Soroti, Uganda; Kenya Medical Research Institute (KEMRI)–Wellcome Trust Research Programme, Kilifi, Kenya; Epicentre, Paris, France; London School of Hygiene and Tropical Medicine, London, UK; Médecins Sans Frontières, Geneva, Switzerland; Médecins Sans Frontières, Geneva, Switzerland; Medical Research Council, Clinical Trials Unit at University College London, London, UK; Médecins Sans Frontières, Geneva, Switzerland; Epicentre, Paris, France; Medical Research Council, Clinical Trials Unit at University College London, London, UK

## Abstract

**Background:**

International guidelines for treatment of children with severe acute malnutrition advise against giving standard oral rehydration solutions (ORS) for dehydration secondary to diarrhoea. Instead, they recommend exclusive use of low-sodium rehydration solution for malnutrition (ReSoMal), due to concerns about both sodium and fluid overload. Supportive evidence is lacking, warranting reappraisal of this guidance. We aimed to assess the safety and superiority of standard WHO-ORS versus ReSoMal.

**Methods:**

GASTROSAM was a phase 2, factorial, open-label, superiority randomised controlled trial conducted at six hospitals in four African countries (Kenya, Niger, Nigeria, and Uganda). Children aged 6 months to 12 years with severe acute malnutrition who were admitted to hospital with severe (stratum A) or moderate (stratum B) dehydration and diarrhoea were randomly assigned in a 1:1 ratio to receive ReSoMal or low-osmolarity WHO-ORS. A simultaneous randomisation in stratum A compared two intravenous strategies versus an oral control rehydration strategy (results reported elsewhere). Children with severe dehydration (stratum A) received their allocated ORS as soon as possible. The primary endpoint was change in sodium concentration at 24 h from baseline in all randomised participants and analysed on an intention-to-treat basis. Children with lived experience were not involved in the study design. The trial is registered on the ISRCTN registry (ISRCTN76149273) and the Pan-African Clinical Trials Registry (PACTR202103852542919).

**Findings:**

Between Sept 2, 2019, and Oct 27, 2024, 415 eligible children were enrolled (218 [53%] male; 197 [47%] female); 272 were enrolled into stratum A (137 to ReSoMal and 135 to WHO-ORS) and 143 were enrolled into stratum B (69 to ReSoMal and 74 to WHO-ORS). Children were followed up for 28 days; 11 (3%) were lost to follow-up or withdrew. The primary endpoint was assessed in 387 (93%) of 415 participants. The increase in sodium concentration was similar in both groups (5·3 mmol/L [SD 8·1] with ReSoMal *vs* 5·0 mmol/L [7·6] with WHO-ORS; mean difference for WHO-ORS *vs* ReSoMal –0·6 [95% CI –1·9 to 0·7], p=0·37). There was no difference in day 28 mortality between the WHO-ORS and ReSoMal groups (19 [9%] *vs* 24 [12%]; adjusted hazard ratio 0·76 [95% CI 0·41 to 1·41], p=0·39). Fluid overload events (pulmonary oedema and cardiac overload) were actively monitored: none was observed.

**Interpretation:**

WHO-ORS resulted in similar outcomes to ReSoMal, and neither strategy led to fluid overload. This finding informs the simplification of guidelines supporting the use of WHO-ORS for the management of dehydration in children regardless of nutritional status.

**Funding:**

Joint Global Health Trials Scheme of the UK Medical Research Council, UK Department for International Development, Wellcome, and Médecins Sans Frontières.

## Introduction

A report from the Global Burden of Diseases, Injuries, and Risk Factors Study in 2019 indicated that diarrhoea was the fifth leading cause of death in children younger than 5 years, resulting in approximately half a million deaths globally.^1^ Some of the highest case fatality rates were recorded in sub-Saharan Africa. Data from the Global Enteric Multicenter Study (GEMS) found that, compared to children with no diarrhoea (controls), children with moderate-to-severe diarrhoea (cases) had an 8·5-times increased risk of dying during the study, and the risk was greatest in those with poor nutritional status.^2^ Severe acute malnutrition is a common cause of hospital admission in African children; however, outcomes including mortality remain poor.^3^ Many cases are complicated with diarrhoea and dehydration, which are both associated with an increased in-hospital mortality risk (27–41%).^4–6^ The current guidance for management of children with severe acute malnutrition who have signs of moderate or severe dehydration secondary to diarrhoea differs from that for children without malnutrition.^7–9^ Management focuses on the exclusive use of oral rehydration with low-sodium oral rehydration solutions (ORS) called rehydration solution for malnutrition (ReSoMal); in 2023, WHO recommended WHO-ORS when ReSoMal is unavailable.^10^ For children with severe dehydration, intravenous rehydration is not recommended and intravenous fluid boluses are only recommended in the presence of shock.^7,11^ The rationale behind this guidance is that children with severe acute malnutrition are considered to be at high risk of cardiac compromise and sodium overload.^11,12^ Similar concerns underpin the rationale for avoiding the use of standard ORS, on the basis that the sodium content is too high. Although such guidance documents have been in place for two and a half decades, no previous^13^ or subsequent evidence has been provided to support these recommendations.^14,15^ Furthermore, the guidance cautions against use of ReSoMal in the community and recommends that it should only be administered under strict medical supervision in a medical facility. Close monitoring at the bedside is advised to check for signs of fluid overload or heart failure, which is difficult to implement in overcrowded paediatric wards with few personnel. This caution might account for the poor outcomes observed in children with severe malnutrition admitted to hospital with diarrhoea and with no pre-referral rehydration as a result of this guidance.

We recently appraised, in an open-label factorial randomised controlled trial, the management of severe dehydration in children with severe malnutrition complicated by diarrhoea, providing evidence to support the safety of intravenous rehydration,^16^ which was also previously demonstrated in a small trial in Bangladesh.^17^ As part of the follow-on management of severe dehydration and immediate management of moderate dehydration in children admitted to hospital with severe acute malnutrition, we also aimed to investigate optimal oral rehydration strategies. We hypothesised that standard WHO low-osmolarity ORS (referred to as WHO-ORS from hereon) would be as effective as the lower-sodium-containing ORS ReSoMal, with fewer side-effects. The comparison of intravenous rehydration has been reported separately.^16^ Here, we report on the oral rehydration comparison, with a primary comparison of change in sodium concentration at 24 h from baseline.

## Methods

### Study design and participants

Gastroenteritis in Severe Malnutrition (GASTROSAM) was a phase 2, open-label, superiority, factorial randomised controlled trial conducted at six hospitals in Uganda (n=2), Kenya (n=2), Niger (n=1), and Nigeria (n=1). The study protocol^18^ and the statistical analysis plan are provided in appendix 1. Children and caregivers with lived experience were not involved in the study design.

Eligible children were those aged 6 months to 12 years admitted to hospital with severe acute malnutrition (defined as either weight-for-height Z score <–3, mid-upper-arm circumference [MUAC] <11·5 cm, or presentation with oedematous malnutrition [kwashiorkor or nutritional oedema] with bilateral pedal or more generalised oedema^7^) with gastroenteritis (>3 loose stools per day) and signs of dehydration. Severe dehydration was defined according to WHO criteria^9^ as two or more of the following: altered consciousness (less than alert on the four-component AVPU scale [with “alert” indicating that the patient is aware and can respond independently, “verbal” indicating that the patient responds only to verbal stimuli, “pain” indicating that the patient responds only to a painful stimulus, and “unresponsive” indicating that the patient does not respond to verbal or painful stimuli]), sunken eyes, reduced skin turgor (slow abdominal skin pinch return >2 s), or inability to take or retain oral fluids. Some or moderate dehydration was defined according to WHO criteria^9^ as two or more of the following signs: restless or irritable, thirsty, sunken eyes, or the skin pinch going back less slowly (≤2 s). Children with known congenital or rheumatic heart disease or with chronic diarrhoea (lasting >14 days) were excluded. When written consent from parents or legal guardians could not be obtained, ethics committees approved verbal assent with delayed written informed consent as soon as practical.^19^ Otherwise, written informed consent was obtained from parents or guardians before randomisation.

This study was conducted in accordance with local regulations, the Declaration of Helsinki, International Ethical Guidelines of the Council of International Organizations of Medical Sciences, and Good Clinical Practice guidelines of the International Conference for Harmonization. The protocol^18^ was approved by independent local and national ethics committees, as well as the ethics committees of Imperial College London (sponsor) and Médecins Sans Frontières (appendix 1 p 9). The protocol had some minor amendments (on June 14, 2019, and Sept 23, 2019) and one major amendment (on Nov 25, 2022) when the Niger and Nigeria sites were added (appendix 1 p 8). No changes were made to the eligibility criteria.

An independent safety data monitoring committee reviewed data by group during the study (across four meetings: June 11, 2020; Dec 12, 2022; May 23, 2024; and Sept 5, 2024; appendix 1 pp 10–11). The trial was registered on the ISRCTN registry (ISRCTN76149273; date registered: Aug 8, 2018) and the Pan-African Clinical Trials Registry (PACTR202103852542919; date registered: March 23, 2021). The trial protocol and statistical analysis plan are posted on ISRCTN76149273.

### Randomisation and masking

The statistician in London (RC) generated the sequential randomisation list, computer-generated with variably sized permuted blocks stratified by site and strata. Randomisation at sites used consecutively numbered opaque sealed envelopes containing the randomised allocation, opened in numerical order. This method was used to ensure that acutely unwell children could be treated immediately at the emergency ward, without having to rely on a computer or the internet to obtain a randomised allocation. An open-label design was used since it was not possible to develop masked interventions for clinicians. Laboratory personnel were masked to the intervention group.

The trial had two strata. Stratum A enrolled children with severe dehydration and stratum B enrolled children with moderate dehydration.

In stratum A, the first factorial randomisation investigated two strategies of intravenous rehydration (rapid or slow) versus control (no intravenous rehydration except in cases of shock). The details of randomisation in stratum A are covered in appendix 1 (pp 6, 23). At the time of randomisation to the intravenous fluid strategy in stratum A, children were simultaneously factorially randomly assigned to receive one of two oral rehydration management strategies, ReSoMal or WHO-ORS, in a 1:1 ratio, once they were able to tolerate enteral fluids. In those receiving intravenous rehydration, ReSoMal or WHO-ORS was started after the intravenous rehydration was complete for children who had persistent clinical evidence of moderate dehydration (see below for volumes and rates). In all cases, per WHO guidelines,^7^ therapeutic milk (F-75 or F-100) was introduced as soon as tolerated, and was counted in the overall oral intake volume.

In stratum B, eligible children with moderate dehydration were randomly assigned (1:1) to receive either ReSoMal or WHO-ORS. Children randomly assigned to receive ReSoMaL (irrespective of age) received 5 mL/kg of ReSoMal every 30 min for the first 2 h then 5–10 mL/kg per h ReSoMal alternated hourly with F-75 up to a maximum of 10 h. Children (irrespective of age) randomly assigned to the WHO-ORS group received 75 mL/kg orally over 4 h, followed by milk feeds. Further doses of WHO-ORS 10 mL/kg were given after each bout of diarrhoea. In all cases, once the child progressed to only mild dehydration (WHO Plan A),^9^ if diarrhoea continued, WHO-ORS (10 mL/kg) or ReSoMal (5 mL/kg) was administered after each episode of diarrhoea, and F-75 milk feed was given every 3 h per standard therapeutic feeding practice. Details of the rehydration solution contents are included in appendix 1 (p 11).

### Procedures

All children with severe acute malnutrition admitted to hospital with an acute history of gastroenteritis were screened for inclusion by study staff. In Niger and Nigeria, participants were transferred to a high dependency ward (assisted ventilation was not available) for management by a dedicated study team. In Uganda and Kenya, participants were managed on general paediatric wards. A structured clinical case report form was completed at admission, documenting sex (on parental report) and salient clinical findings. Race or ethnicity data were not collected.

Basic infrastructural support for emergency care, patient monitors, bedside haemoglobin, and glucose and lactate point-of-care tests were provided by the study to hospitals. For children in both strata, bedside observations were performed at admission and every 30 min for the first 2 h, hourly up to 8 h, then at 12, 24, 36, and 48 h after randomisation. Clinical chemistry was assessed at 0, 8, and 24 h. Blood cultures were performed in sites where microbiological facilities were available (except for Mombasa, where microbiological facilities were not available). Participants who were unable to tolerate oral fluids had a nasogastric tube placed to administer oral rehydration fluids and F-75. At each administration of oral rehydration the positioning of the nasogastric tube was checked to ensure safety. At each of the clinical reviews, children were actively monitored for serious adverse events, particularly clinical signs of suspected cardiac or pulmonary overload. Clinicians were taught specifically on how to elicit these signs and treat complications such as hypoglycaemia. Participants were clinically assessed at 7 days (including clinical chemistry) and 28 days (trial exit) post-randomisation. Study staff were unmasked throughout; laboratory tests were assayed masked.

### Outcomes

The primary endpoint was change in sodium concentration at 24 h from baseline. Secondary efficacy endpoints were change in sodium concentration at 8 h, mortality to day 28, and change in bodyweight and MUAC at day 3 and day 7. Safety endpoints were evidence of pulmonary oedema or heart failure, change in sodium at 24 h compared with 8 h (post-intravenous strategy completion), and correction of electrolyte abnormalities (severe hyponatremia [plasma sodium concentration <125 mmol/L] or hypokalaemia [plasma potassium concentration <2·5 mmol/L]).

### Statistical analysis

Sample size calculations were based on the intravenous rehydration comparison primary endpoint of mortality in 272 children with severe dehydration; 143 children with moderate dehydration enrolled into stratum B were included in this comparison of ORS. 415 eligible children resulted in more than 90% power to detect a 3 mmol/L difference in sodium concentrations (assuming a mean of 132 mmol/L and SD of 7) at 24 h between the ReSoMal and WHO-ORS groups with a greater anticipated increase in the WHO-ORS group. Severe hyponatraemia events were not anticipated to be high enough for a primary binary outcome so a suitable continuous outcome was used (absolute difference in sodium; appendix 1 p 7).

Randomised groups were compared with an intention-to-treat analysis. Continuous outcomes were analysed with linear regression adjusted for site, strata, and baseline values to estimate the difference in means. Binary outcomes were compared with Mantel–Haenszel odds ratios or p values from Fisher’s exact test where there were zero events in a category. Mortality at 28 days was estimated by hazard ratios from Cox regression adjusted for site and strata, and subhazard ratios for time to correction of hyponatraemia and hypokalaemia were calculated with competing risks regression adjusted for site and strata, taking into account death. 95% CIs were calculated for each estimate of the treatment effect. Complete case analyses are presented for primary and secondary endpoints following the statistical analysis plan under a missing completely at random assumption. Subgroup analysis was done for the primary endpoint using tests of interaction for the following prespecified subgroups: intravenous comparison for those with severe dehydration, age (<1 year or ≥1 year), consciousness level, respiratory distress at randomisation, HIV status, and strata.

### Role of the funding source

The Joint Global Health Trials Scheme of the UK Medical Research Council, UK Department for International Development, and Wellcome had no role in trial design, data collection, data analysis, data interpretation, or writing of the report. Médecins Sans Frontières co-investigators were involved in trial design, data collection, data analysis, data interpretation, and writing of the report.

## Results

**Figure 1:**
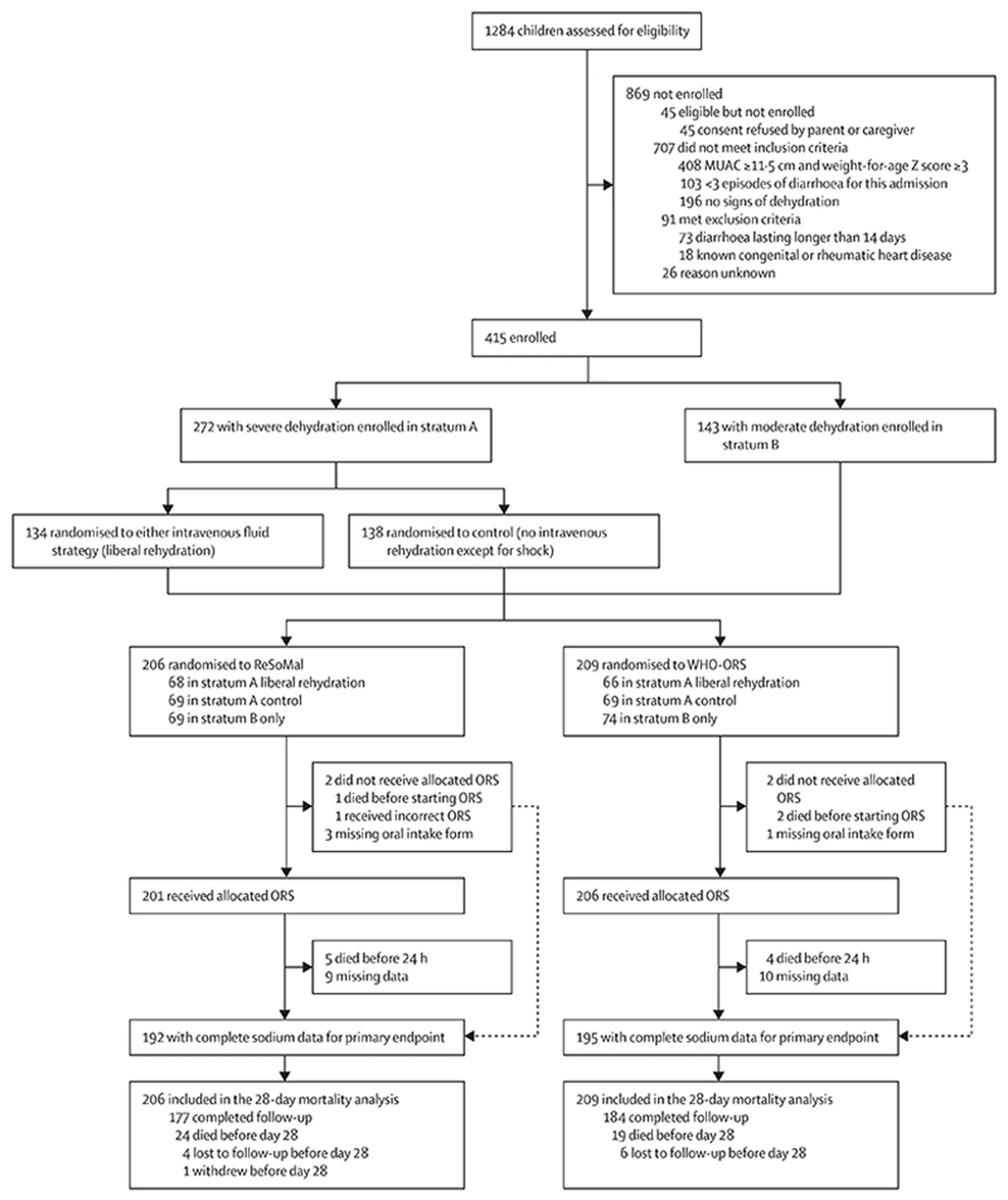
Trial profile MUAC=mid-upper-arm circumference. ORS=oral rehydration solution. ReSoMal=rehydration solution for malnutrition.

Between Sept 2, 2019, and Oct 27, 2024, 415 eligible children were enrolled: 272 children with severe dehydration were randomly assigned to stratum A and 143 children with moderate dehydration were randomly assigned to stratum B. Overall, across the two strata, 206 children were allocated to ReSoMal and 209 were allocated to WHO-ORS. All children were included in the intention-to-treat analysis (figure 1). Recruitment was halted between March 23, 2020, and Dec 5, 2021, due to the COVID-19 pandemic. Baseline characteristics are presented for the combined groups in both strata (table 1) and separately by group for each stratum (appendix 1 pp 12–15). Five children eligible for assignment to stratum A were incorrectly assigned to stratum B (all had slow abdominal skin pinch return >2 s [decreased skin turgor]). The median age was 13 months (IQR 9–23) and similar in the two strata. Overall, the median weight-for-height Z score was –4·7 (IQR –5·5 to –4·0) and was lower in stratum A than in stratum B. Few children had nutritional oedema (24 [6%] of 415). Although, by design, features indicating severe dehydration and impaired perfusion were uncommon in stratum B, the frequency of various laboratory measures was similar in both strata at baseline, including the proportion of children with hypoglycaemia (overall 36 [9%] of 408), the proportion of children with malaria rapid diagnostic test (RDT) positivity (overall 71 [17%] of 415), and the proportion of children with bacteraemia (overall 20 [12%] of 173). No child included in the trial was diagnosed with cholera. The numbers of children with severe hyponatraemia (sodium <125 mmol/L: 45 [32%] of 139) and severe hypokalaemia (potassium <2·5 mmol/L: 38 [27%] of 139) at baseline were less frequent in stratum B than in stratum A (appendix 1 p 14). Overall, 222 (54%) of 414 parents reported that their child had already received some ORS during this period of illness and 199 (49%) of 404 reported that their child had been admitted to another health facility before admission to the trial site hospital (table 1).

All children received their allocated oral rehydration by randomisation (table 2) except for three patients (two in the WHO-ORS group and one in the ReSoMal group who died before starting their allocated ORS strategy) and one patient who was randomly assigned to ReSoMal but received WHO-ORS. Median time to start the ORS (from randomisation) was overall slightly longer in stratum A (1·4 h [IQR 0·3–7·7]) than stratum B (0·3 h [0·2–0·6]; appendix 1 p 16). However, this reflects that half the patients in stratum A were receiving intravenous rehydration (over 4–8 h per intravenous strategy) initially before commencing oral rehydration (appendix 1 p 16). The volumes of received fluids followed the recommendations in the trial protocol for both strata (table 2; appendix 1 p 16). Overall, the ReSoMal group received a lower volume of fluids within 24 h of randomisation (median 90 mL/kg [IQR 66–120]) compared to the WHO-ORS group (median 135 mL/kg [95–180]) over the same time period (table 2). By 4 h, the median volume received was 30 mL/kg (IQR 26–40) in the ReSoMal group compared to 75 mL/kg (66–76) in the WHO-ORS group (table 2). For stratum B, in the intention-to-treat analysis, the ReSoMal group also received a lower volume of fluids than the WHO-ORS group within 24 h (median 81 mL/kg [IQR (70–110] *vs* 125 mL/kg [96–152]; appendix 1 p 17). Similar volumes of liquid feed (F-75) were given in both groups; however, as indicated above, ORS was started only after intravenous rehydration (median 5·3 h [IQR 4·0–6·9] for the liberal rapid intravenous rehydration strategy and 8·7 h [IQR 8·4–9·2] for the liberal slow intravenous rehydration strategy; appendix 1 pp 6, 16).

**Figure 2:**
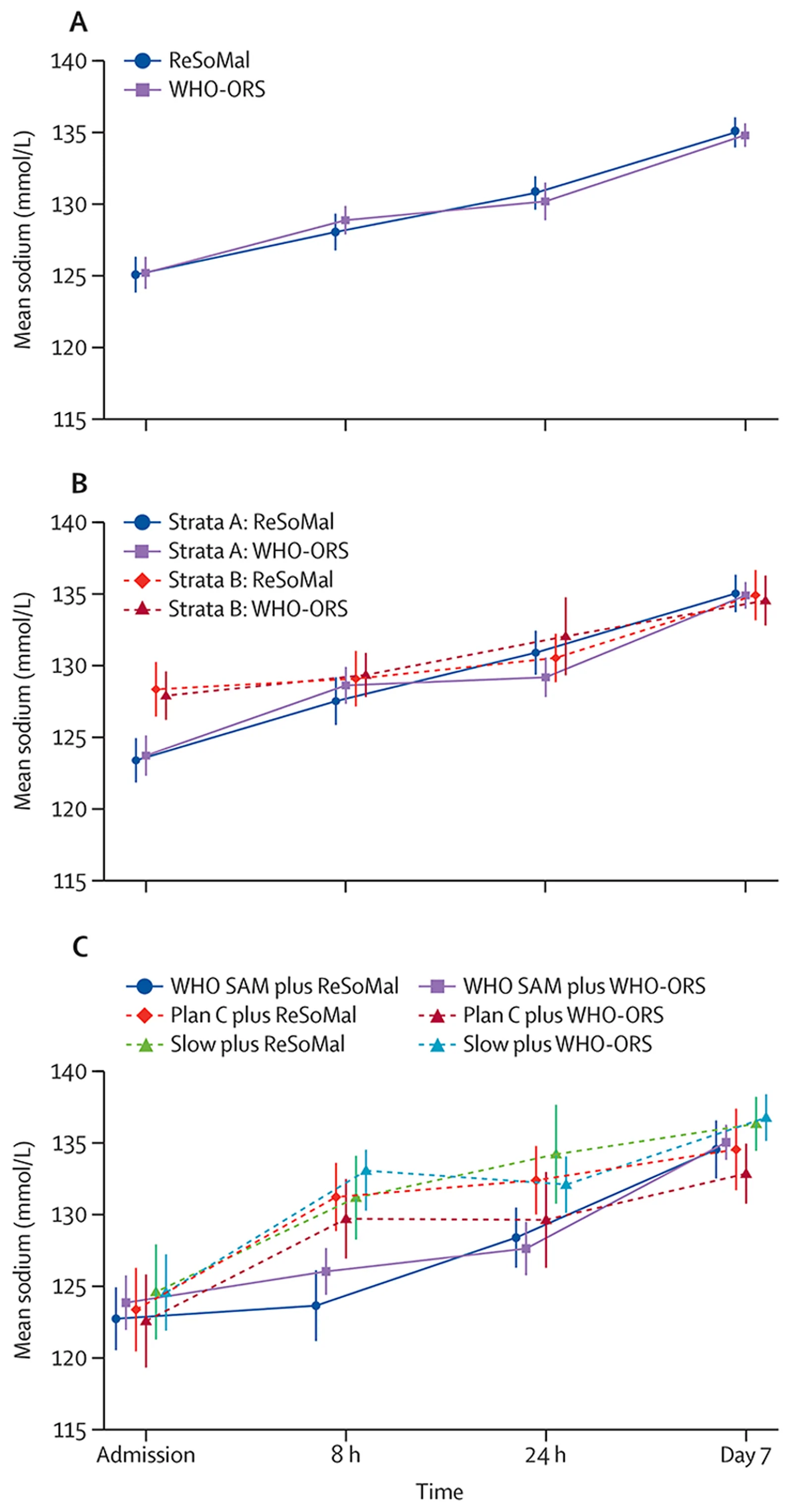
Mean sodium over time (A) By ORS randomisation. (B) By strata and ORS randomisation. (C) By intravenous and ORS randomisation in strata A only. Error bars represent 95% CIs. ORS=oral rehydration solution. ReSoMal=rehydration solution for malnutrition. SAM=severe acute malnutrition.

**Figure 3:**
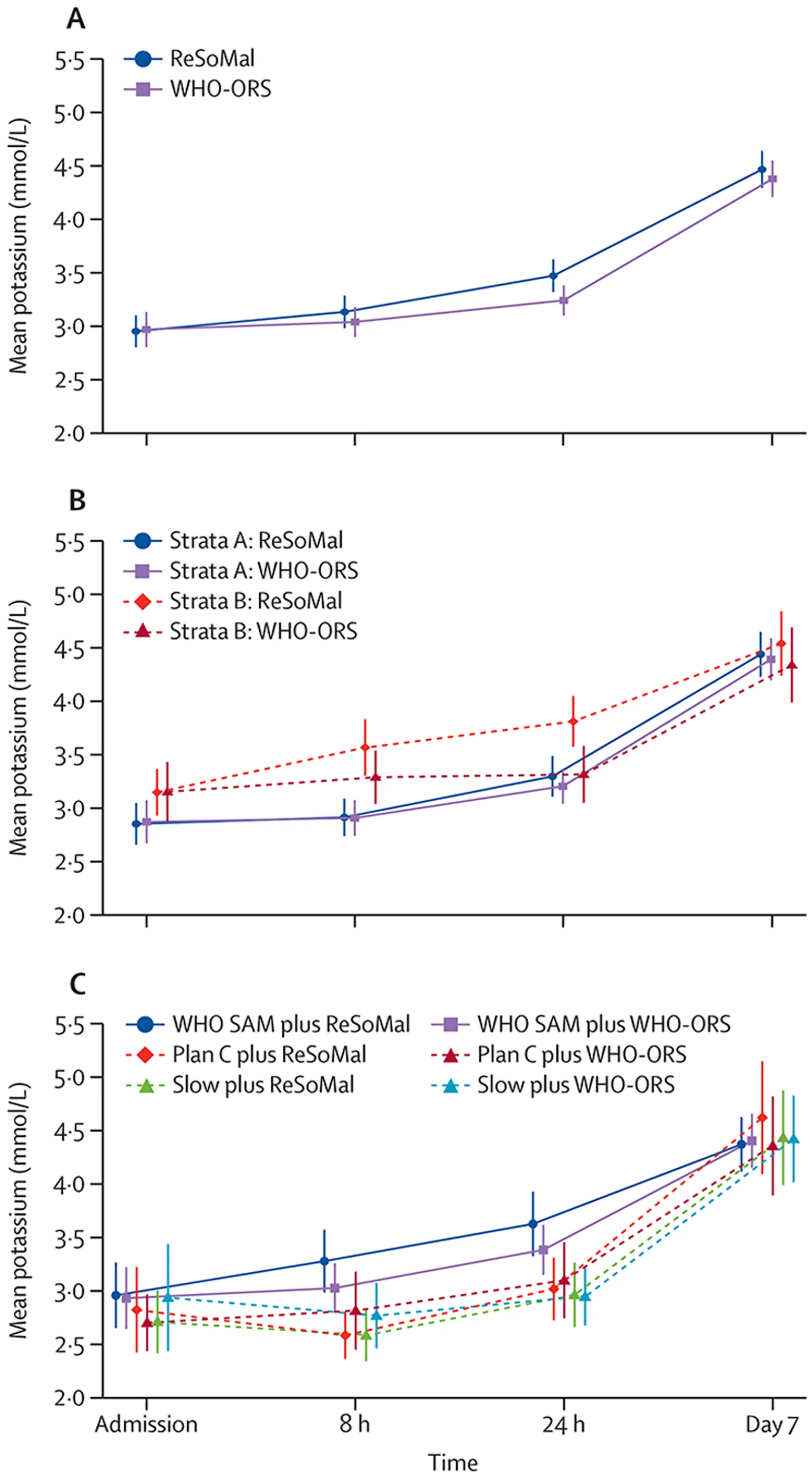
Mean potassium over time (A) By ORS randomisation. (B) By strata and ORS randomisation. (C) By intravenous and ORS randomisation in strata A only. Error bars represent 95% CIs. ORS=oral rehydration solution. ReSoMal=rehydration solution for malnutrition. SAM=severe acute malnutrition.

The primary endpoint, change in sodium concentration at 24 h, was available in 192 (93%) of 206 children in the ReSoMal group and 195 (93%) of 209 children in the WHO-ORS group, with missing samples due to a combination of either death before 24 h (five children in the ReSoMal group and four in the WHO-ORS group) or missing samples at one or both timepoints (figure 1; appendix 1 pp 17–19). Thus, after exclusion of deaths before 24 h, samples were available for the primary endpoint in 387 (95%) of 406 participants. There was no significant difference in the change in sodium concentration between the two groups (5·3 mmol/L [SD 8·1] with ReSoMal *vs* 5·0 mmol/L [7·6] with WHO-ORS; mean difference for WHO-ORS *vs* ReSoMal –0·6 [95% CI –1·9 to 0·7; p=0·37; table 3). At 8 h, severe hyponatraemia was significantly more common in the ReSoMal group (63 [32%] of 198) than in the WHO-ORS group (42 [22%] of 194; p=0·023). By 24 h, fewer children had severe hyponatraemia (present in 79 [20%] of 391 children overall), with no difference between the groups. At 8 h the presence of severe hypokalaemia was similar in both groups (present in 128 [33%] of 390 children overall), and the change in potassium was marginally greater in the ReSoMal group than in the WHO-ORS group (median 0·2 mmol/L [SD 0·9] *vs* 0·1 mmol/L [0·9]; mean difference –0·1 [95% CI –0·3 to 0·0]; p=0·093). By 24 h, there was no difference in the numbers of children with severe hypokalaemia in both groups (overall 82 [21%] of 388 children), and the increase in plasma potassium was marginally greater in the ReSoMal group than in the WHO-ORS group (median 0·5 mmol/L [SD 0·8] *vs* 0·3 mmol/L [1·0]; mean difference –0·3 (95% CI –0·4 to –0·1; p=0·0024). Time to correction of severe hyponatraemia and severe hypokalaemia was similar between the study groups. Development of hyper-natraemia (sodium >145mmol/L) was rare (14 [4%] of 391 children by 24 h), with no difference between the groups. By day 7, most severe perturbations of sodium and potassium had largely resolved (figures 2, 3). At day 3 and day 7 the changes in all anthropometric parameters were similar in both groups (table 3).

Findings for the primary outcome were consistent across six prespecified subgroups: intravenous comparison for those with severe dehydration, age (<1 year or ≥ 1 year), consciousness level, respiratory distress at randomisation, HIV status, and between strata (appendix 1 p 22).

Separate analyses for each study stratum are presented in appendix 1 (pp 18–21). Salient findings include the fact that the prevalence of severe hyponatraemia was more common at 8 h in stratum A with ReSoMal than with WHO-ORS (48 [37%] of 131 *vs* 30 [24%] of 126; p=0·030) but was similar at 24 h (28 [22%] of 128 in both groups). Although the change in sodium concentration at 24 h from post-intravenous concentrations (8 h) was significantly higher in the ReSoMal group than in the WHO-ORS group (2·9 mmol/L [SD 6·6] *vs* 0·6 mmol/L [5·9]; mean difference –2·3 (95% CI –3·6 to –0·9; p=0·0013; appendix 1 p 18), this was possibly due to the higher prevalence of severe hyponatraemia at 8 h in the ReSoMal group, as reported above. Among children directly enrolled with moderate dehydration (stratum B), there was no difference in change in plasma sodium at 24 h (2·2 mmol/L [SD 7·2] *vs* 3·6 mmol/L [8·4]; mean difference 0·9 [95% CI –1·4 to 3·2]; p=0·43; appendix 1 p 20); nor were there any differences in the frequencies of severe hyponatraemia over prespecified timepoints. Additionally, the change in potassium at 8 h was greater in the ReSoMal group than in the WHO-ORS group (0·4 [SD 0·9] mmol/L *vs* 0·1 [0·9] mmol/L; mean difference –0·3 [95% CI –0·6 to –0·1]; p=0·013; appendix 1 p 20).

Vital status at day 28 was known for 404 (98%) of 415 children, with four (2%) and six (3%) children lost to follow up in the ReSoMal and ORS groups respectively, and one withdrawal in ReSoMal group. Overall, the prespecified day 28 mortality secondary endpoint was similar in the two groups: 24 (12%) with ReSoMal versus 19 (9%) with WHO-ORS (adjusted hazard ratio 0·76 [95% CI 0·41–1·41]; p=0·39). A post-hoc analysis of mortality at day 3 and day 7 found no difference between the groups at either timepoint (appendix 1 p 22). A separate analysis for stratum B also found that day 28 mortality was similar between respective study groups (six [9%] of 69 with ReSoMal *vs* six [8%] of 74 with WHO-ORS; hazard ratio 0·85 [95% CI 0·27–2·63]; p=0·77; appendix 1 pp 20–21). In stratum B, day 3 mortality in the ReSoMal versus WHO-ORS groups was five (7%) versus two (3%), respectively, and day 7 mortality was six (9%) versus five (7%), respectively (appendix 1 p 23).

Although actively clinically monitored for throughout the trial, none of the 415 children developed pulmonary oedema or signs consistent with heart failure in the either strata or groups.

## Discussion

In this randomised controlled trial conducted in four African countries, which enrolled children with severe acute malnutrition complicated by moderate and severe dehydration due to gastroenteritis, we found that WHO-ORS resulted in similar outcomes to the currently recommended ReSoMal. Importantly, at 8 h, fewer children receiving WHO-ORS had severe hyponatraemia (sodium <125 mmol/L) than did those receiving ReSoMal. The increase in plasma potassium in the ReSoMal group was only marginally superior (absolute difference of 0·1 mmol/L at 8 h and 0·3 mmol/L at 24 h), which corresponds with the higher potassium content of ReSoMal. However, this observation has minimal implications considering the normal range of potassium is 3·5–5·0 mmol/L, and the median in this trial was 2·8 mmol/L at baseline. There was no evidence of a difference in mortality at day 28 between the groups. Although we actively monitored for clinical features consistent with fluid overload or cardiac failure, none was found, including in children receiving oral rehydration following liberal intravenous rehydration (100 mL/kg).

Key strengths of this trial include the multicentre design, incorporating children from areas with seasonal malnutrition (Niger and Nigeria) as well as children from populations where malnutrition is endemic in the communities (Uganda and Kenya). The high adherence to randomised ORS and to the volumes (and rates) required by the trial protocol, as well as the high retention rates for the primary endpoint (93% rising to 95% when deaths before this endpoint were excluded) and vital status at day 28 (98%) indicate that these results have high internal validity and minimal bias. The finding of no events of fluid overload and similar mortality are reassuring and could lead to a re-thinking of current recommendations against the use of oral hydration solutions for children with severe malnutrition in community settings. Providing ORS in the community to children with severe acute malnutrition with less severe degrees of dehydration might prevent hospital admission and the poor outcomes reported previously.^3–5,20^

In this trial, a higher proportion of children receiving ReSoMal had severe hyponatraemia (at 8 h) than those receiving WHO-ORS. Even though the proportions were similar at 24 h, the risk of adverse outcomes is highest in the first 8 h post-admission. This is a concern since severe hyponatraemia has been demonstrated as an independent risk factor for early mortality.^3^ Our sample size assumed only a modest difference in sodium (3 mmol/L) between the intervention (WHO-ORS) and control (ReSoMal) groups. As highlighted in appendix 1 (pp 6–7), the available data on sodium correction before the design of GASTROSAM were very sparse, with no data available from the African continent to underpin an estimated difference for sample size. Our key concern, which underpinned our hypothesis, was that development of severe hyponatraemia, secondary to rehydration with ReSoMal, was too infrequently demonstrated in previous observational studies to justify as an endpoint.^21,22^ Thus, we focused on sodium change as a continuous measure, which was measured by laboratory staff masked to the intervention group (thus minimising the risk of bias), and safety endpoints.

One potential trial limitation was that we did not include children or their caregivers with lived experience in the research group. A second potential limitation was that while we found no evidence of a difference in mortality, as noted previously^16^ the mortality rates in this trial were considerably lower than those observed in children receiving routine care.^4,5^ A key reason for the low mortality in our trial might be that most children (90%) were managed on high dependency wards by dedicated clinical trial teams and received close and frequent monitoring to identify and treat complications (specifically fluid overload, shock, or hypoglycaemia) and to ensure protocol adherence. These measures were put in place to address concerns by ethics committees about the balance of risk to benefit for children participating in the trial. These concerns were due to longstanding international guidance and teaching against the use of intravenous rehydration and extremely cautious use of oral rehydration in children with severe acute malnutrition due to the perceived risk of heart failure and volume overload.^7,9^ This raises questions about generalisability since the level of care and attention to patient safety in our trial might not be translatable to under-resourced and overcrowded paediatric wards in African countries. Nevertheless, if we had conducted the trial without additional research staff and attention to patient safety this might have led to poor adherence to the randomised intervention as well as incomplete ascertainment of the primary and critical safety events. The data generated would have been of low quality, wasting participant involvement and yielding few or misleading conclusions. By contrast, rigorously conducted trials provide robust new evidence that is both credible and reliable.

The GASTROSAM trial provides data supporting the safe use of WHO-ORS as an option for rehydration, which is the first step in the review of current guidelines. Although the validity of the signs of dehydration in children with severe acute malnutrition remains controversial^10^ the trial was designed to appraise both intravenous and oral rehydration and followed the definitions of severe and moderate dehydration (for children with severe acute malnutrition and children without severe acute malnutrition) indicated in the WHO guidelines for the management of common childhood illnesses.^8^ Due to the randomised design, any misclassification of dehydration status would be the same across all groups.

A systematic review of oral rehydration conducted by Houston and colleagues^15^ in 2017 identified only two small clinical trials conducted in Asian children that compared the use of ReSoMal against other types of ORS;^21,22^ both trials showed that the development of hyponatraemia was more frequent with ReSoMal than with the other ORS. In the first trial, three children developed seizures associated with severe hyponatraemia.^21^ The trial compared ReSoMal with hypo-osmolar ORS and found that a significantly greater proportion of children in the ReSoMal group developed hyponatraemia (15·4% *vs* 1·9%, p=0·03).^22^ Our study therefore adds to this literature on the potential harm of exposing children to the development of severe hyponatraemia or exacerbating pre-existing deficits with ReSoMal.

Collectively, the existing scarce evidence in Africa together with new knowledge provided by the GASTROSAM trial show that more liberal intravenous^16^ and oral rehydration strategies are safe. As a result, standard WHO low-osmolarity ORS has emerged as a viable option to treat dehydration both within and possibly outside of hospitals. These data should now be used to close the therapeutic gap specifically in settings where ReSoMal is not in stock, which is a frequent occurrence in hospitals in Africa. It could be argued that ReSoMal should be retained since it contains additional electrolytes that could be beneficial to a malnourished child. However, this is largely irrelevant since these electrolytes are already included in the nutritional milks (and in nutritional ready-to-use feeds). Moreover, our study demonstrated that ReSoMal provided a superior but clinically negligible improvement in potassium concentrations, which should not preclude the consideration of low-osmolarity WHO-ORS as a recommended treatment option.

In conclusion, we found that standard low-osmolarity WHO-ORS was a safe option for rehydration in children with severe acute malnutrition and dehydration and we suggest that current guidance be reviewed to consider simplifying rehydration protocols, removing the distinction in management between children with severe acute malnutrition and non-malnourished children, which would be more pragmatic in the underresourced settings where most children are managed. These data could also pave the way for reconsidering whether oral rehydration can be started in the community in children with severe acute malnutrition, which is currently not recommended owing to the potential risk of fluid and sodium overload.

## Supplementary Material

S1

Appendix 1

## Figures and Tables

**Table 1:** Baseline characteristics

	ReSoMal (n=206)	WHO-ORS (n=209)	Total (N=415)
Site			
Mbale, Uganda	10 (5%)	10 (5%)	20 (5%)
Soroti, Uganda	10 (5%)	8 (4%)	18 (4%)
Kilifi, Kenya	12 (6%)	12 (6%)	24 (6%)
Mombasa, Kenya	1 (0%)	4 (2%)	5 (1%)
Maiduguri, Nigeria	80 (39%)	81 (39%)	161 (39%)
Magaria, Niger	93 (45%)	94 (45%)	187 (45%)
Sex			
Male	107 (52%)	111 (53%)	218 (53%)
Female	99 (48%)	98(47%)	197(47%)
Age, months	14 (9 to 24)	12 (9 to 21)	13 (9 to 23)
Bodyweight (kg)	5·6 (4·8 to 6·4)	5·3 (4·5 to 6·2)	5·5 (4·6 to 6·3)
Weight-for-height Z score	–4·6 (–5·5 to –3·9)	–4·8 (–5·6 to –4·2	–4·7 (–5·5 to –4·0)
MUAC (cm)	10·5 (9·8 to 11·1); n=204	10·3 (9·5 to 11·0); n=209	10·6 (9·5 to 11·0); N=413
Nutritional oedema	11 (5%)	13 (6%)	24 (6%)
Altered consciousness*	53 (26%)	51 (24%)	104 (25%)
Restless or irritable	82 (40%)	90 (43%)	172(41%)
Sunken eyes	200 (97%)	206 (99%)	406 (98%)
Abdominal skin pinch 1–2 s	72 (35%)	79 (38%)	151 (36%)
Abdominal skin pinch >2 s	126 (61%)	118 (56%)	244 (59%)
Unable to take or retain oral fluids	109 (53%)	110 (53%)	219 (53%)
Fever (temperature >375°C)	75/206 (36%)	68/208 (33%)	143/414 (35%)
Severe tachypnoea (>40 breaths per min)	59 (29%)	57 (27%)	116 (28%)
Oxygen saturation (%)	100 (98 to 100); n=206	100 (98 to 100); n=208	100 (98 to 100); N=414
Heart rate (beats per min)	137 (119 to 156)	138 (122 to 156)	138 (120 to 156)
Severe tachycardia^†^	115 (56%)	107 (51%)	222 (53%)
Capillary refill time >3 s	17 (8%)	23 (11%)	40 (10%)
Weak pulse	26 (13%)	29 (14%)	55 (13%)
Cold peripheries	24/205 (12%)	23/209 (11%)	47/414 (11%)
Moderate hypotension	51/198 (26%)	42/203 (21%)	93/401 (23%)
WHO shock	12 (6%)	13 (6%)	25 (6%)
Indrawing	16/206 (8%)	19/207 (9%)	35/413 (8%)
Deep breathing	13/206 (6%)	20/207 (10%)	33/413 (8%)
Lung crepitations	24/202 (12%)	24/202 (12%)	48/404 (12%)
Bloody diarrhoea	30/206 (15%)	40/206 (19%)	70/412 (17%)
History of vomiting	149/206 (72%)	164/207 (79%)	313/413 (76%)
Appetite (1–10 scale)	3 (2 to 5); n=205	3 (2 to 5); n=206	3 (2 to 5); N=411
Still breastfeeding	95/203 (47%)	109/206 (53%)	204/409 (50%)
Currently on nutritional feed	18/195 (9%)	23/208 (11%)	41/403 (10%)
Already taken oral rehydration solution for this illness	110/206 (53%)	112/208 (54%)	222/414 (54%)
Admitted to another facility in this illness	102/196 (52%)	97/208 (47%)	199/404 (49%)
Diarrhoea in past 6 months	95/204 (47%)	107/208 (51%)	202/412 (49%)
Previous admission for malnutrition	52/196 (27%)	61/207 (29%)	113/403 (28%)
Laboratory measures			
Hypoglycaemia (glucose <3 mmol/L)	17/202 (8%)	19/206 (9%)	36/408 (9%)
Sodium (mmol/L)	125 (120 to 130); n=200	126 (120 to 131); n=201	125 (120 to 131); N=401
Severe hyponatraemia (sodium <125 mmol/L)	94/200 (47%)	88/201 (44%)	182/401 (45%)
Potassium (mmol/L)	2·8 (2·1 to 3·5); n=197	2·8 (2·1 to 3·5); n=200	2·8 (2·1 to 3·5); N=397
Severe hypokalaemia (potassium <2·5 mmol/L)	75/197 (38%)	78/200 (39%)	153/397 (39%)
Chloride (mmol/L)	98 (94 to 105); n=192	99 (94 to 104); n=195	99 (94 to 105); N=387
Bicarbonate (mmol/L)	14 (11 to 18); n=83	14 (11 to 18); n=83	14 (11 to 18); N=166
Living with HIV	5 (2%)	6 (3%)	11 (3%)
Malaria rapid test positive	40 (19%)	31 (15%)	71 (17%)
Haemoglobin (g/dL)	11·1 (9·5 to 12·6); n=198	10·7 (9·4 to 12·2); n=199	10·9 (9·5 to 12·5); N=397
Severe anaemia (haemoglobin <5 g/dL)	1/198 (1%)	2/199 (1%)	3/397 (1%)
Blood culture positive	9/84 (11%)	11/89 (12%)	20/173 (12%)

Data are n (%) or n/N (%) for categorical variables and median (IQR) for continuous variables. MUAC=mid-upper-arm circumference. ORS=oral rehydration solution. ReSoMal=rehydration solution for malnutrition.

* Altered consciousness: the level of consciousness was assessed on the basis of the four-component AVPU scale. Altered level of consciousness was considered to be a status of verbal, pain, or unresponsive.

† Severe tachycardia was defined as a heart rate greater than 160 bpm in children aged younger than 12 months and greater than 120 bpm if children were aged 12 months or older.

**Table 2:** Adherence to randomised strategy and other clinical management (strata A and B combined)

	ReSoMal	WHO-ORS	Total
**ReSoMal (mL/kg)**			
Total	90 (66–120)	··	0 (0–90)
0 to <4 h	30 (26–40)	··	0 (0–30)
4 to <8 h	20 (10–20)	··	0 (0–20)
8 to <16 h	21 (10–40)	··	0 (0–21)
**WHO-ORS (mL/kg)**			
Total	··	135(95–180)	0 (0–135)
0 to <4 h	··	75 (66–76)	0 (0–75)
4 to <8 h	··	15 (0–39)	0 (0–19)
8 to <16 h	··	20 (10–40)	0 (0–20)
**F-75 (mL/kg)**			
N (%) received	198 (98%)	191 (93%)	389 (95%)
Total	114 (87–122)	114 (97–130)	114 (92–128)
0 to <4 h	10 (10–19)	0 (0–17)	10 (0–17)
4 to <8 h	20 (17–20)	17 (16–32)	20 (16–22)
8 to <16 h	36 (31–48)	36 (32–49)	36 (32–49)
**Other management**			
Used nasogastric tube (all)	146/202 (72%)	153/206 (74%)	299/408 (73%)
Strata A only	104/133 (78%)	105/132 (80%)	209/265 (79%)
Strata B only	42/69 (61%)	48/74 (65%)	90/142 (63%)
Number with corrections for hypoglycaemia	11/206 (5%)	18/207 (9%)	29/413 (7%)

Data are n/N (%) or median (IQR). Three children (one in the ReSoMal group and two in the WHO-ORS group) died before starting ORS. Four children (three in the ReSoMal group and one in the WHO-ORS group) had no oral intake form available. ORS=oral rehydration solution. ReSoMal=rehydration solution for malnutrition.

**Table 3:** Outcomes

	ReSoMal (n=206)	WHO-ORS (n=209)	Overall	Estimate of effect,* WHO-ORS *vs* ReSoMal (95% CI)	p value
**Primary outcome**					
Number of children with data	192	195	387	··	··
Change in sodium (mmol/L) at 24 h	5·3 (8·1)	5·0 (7·6)	5·2 (7·8)	–0·6 (–1·9 to 0·7)	0·37
**Secondary outcomes**					
Mortality at 28 days	24 (12%)	19 (9%)	43 (10%)	0·76 (0·41 to 1·41)^†^	0·39
Suspected pulmonary oedema	0 (0%)	0 (0%)	0 (0%)	··	··
Suspected heart failure	0 (0%)	0 (0%)	0 (0%)	··	··
Change in sodium (mmol/L) at 24 h from post-intravenous rehydration (8 h), in stratum A only	2·5 (6·2); n=137	1·2 (6·1); n=135	1·8 (6·2); n=272	–2·3 (–3·6 to –0·9)	0·0013
**Biochemistry**					
8 h					
Severe hyponatraemia (sodium <125 mmol/L)	63/198 (32%)	42/194 (22%)	105/392 (27%)	0·59 (0·37 to 0·94)	0·023
Hypernatraemia (sodium >145 mmol/L)	6/198 (3%)	1/194 (1%)	7/392 (2%)	0·17 (0·02 to 1·41)	0–061
Severe hypokalaemia (potassium <2·5 mmol/L)	63/197 (32%)	65/193 (34%)	128/390 (33%)	1·08 (0·71 to 1·65)	0·72
Change in sodium concentration (mmol/L) at 8 h	3·0 (6·6)	3·7 (6·0)	3·4 (6·3)	0·6 (–0·5 to 1·7)	0·27
Change in potassium (mmol/L) at 8 h	0·2 (0·9)	0·1 (0·9)	0·1 (0·9)	–0·1 (–0·3 to 0·0	0·093
24 h					
Severe hyponatraemia (sodium <125 mmol/L)	40/194 (21%)	39/197 (20%)	79/391 (20%)	0·95 (0·58 to 1·56)	0·84
Hypernatraemia (sodium >145 mmol/L)	8/194 (4%)	6/197 (3%)	14/391 (4%)	0·73 (0·25 to 2·15)	0·57
Severe hypokalaemia (potassium <2·5 mmol/L)	38/192 (20%)	44/196 (22%)	82/388 (21%)	1·17 (0·72 to 1·91)	0·52
Change in potassium (mmol/L) at 24 h	0·5 (0·8)	0·3 (1·0)	0·4 (0·9)	–0·3 (–0·4 to –0·1)	0·0024
7 days					
Severe hyponatraemia (sodium <125 mmol/L)	5/142 (4%)	7/149 (5%)	12/291 (4%)	1·35 (0·42 to 4·37)	0·61
Hypernatraemia (sodium >145 mmol/L)	4/142 (3%)	0/149 (0%)	4/291 (1%)	··	0·055
Severe hypokalaemia (potassium <2·5 mmol/L)	4/139 (3%)	6/149 (4%)	10/288 (3%)	1·42 (039 to 5·14)	0·60
Change in sodium (mmol/L) at day 7 visit	9·3 (8·1)	10·2 (8·3)	9·8 (8·2)	–0·1 (–14 to 14)	0·82
Change in potassium (mmol/L) at day 7 visit^‡^	1·5 (1·2)	1·5 (1·2)	1·5 (1·2)	–0·1 (–03 to 04)	0·51
Time to correction of severe hyponatraemia (subhazard ratio)^§^	··	··	··	1·19 (0·93 to 1·51)	0·17
Time to correction of severe hypokalaemia (subhazard ratio)^§^	··	··	··	1·08 (0·81 to 144)	0·59
**Anthropometry (secondary outcomes)**					
Day 3					
Number of children with data	187	192	379	··	··
Mean change in bodyweight (kg)^‡^	0·4 (0·3)	0·4 (0·3)	0·4 (0·3)	0·0 (–0·0 to 0·1)	0·23
Mean change in weight-for-age Z score^‡^	0·4 (0·3)	0·5 (0·3)	0·4 (0·3)	0·0 (–0·0 to 0·1)	0·31
Mean change in MUAC (cm)^‡^	0·3 (0·4)	0·3 (0·4)	0·3 (0·4)	–0·0 (–0·1 to 0·1)	0·72
Mean change in MUAC-for-age Z score^‡^	0·3 (0·4)	0·3 (0·4)	0·3 (0·4)	–0·0 (–0·1 to 0·1)	0·57
Day 7					
Number of children with data	183	192	375	··	··
Mean change in bodyweight (kg)	0·6 (0·5)	0·6 (0·4)	0·6 (04	0·0 (–04 to 04)	0·99
Mean change in weight-for-age Z score	0·7 (0·5)	0·7 (0·5)	0·7 (0·5)	0·0 (–04 to 04)	0·67
Mean change in MUAC (cm)	0·6 (0·6)	0·6 (0·6)	0·6 (0·6)	0·0 (–04 to 04)	0·98
Mean change in MUAC-for-age Z score	0·6 (0·7)	0·6 (0·7)	0·6 (0·7)	0·0 (–04 to 04)	0·87

MUAC=mid-upper-arm circumference. ORS=oral rehydration solution. ReSoMal=rehydration solution for malnutrition.

* Continuous outcomes summarised with means and SDs, and estimate of effect analysed with linear regression adjusted for site, strata, and baseline values. Binary outcomes are summarised by N (%) and estimate of effects are unadjusted Mantel–Haenszel odds ratios. Where there are 0 children in a group, p values are derived from Fisher’s exact test.

† Hazard ratio from Cox model adjusted for site and strata.

‡ Exploratory outcome, which was prespecified in the protocol, inadvertently not in statistical analysis plan.

§ Time to correction of hyponatraemia and hypokalaemia were calculated with competing risks regression taking into account death, adjusted for site and strata. Exploratory outcome to add information alongside frequency of severe hypokalaemia and hyperkalaemia at this timepoint. Severe hyponatraemia defined as sodium <125 mmol/L. Severe hypokalaemia defined as potassium <2·5 mmol/L. Hypernatraemia defined as sodium >145 mmol/L.

## Data Availability

The data will be made available on reasonable request to ECG 1 year after publication of the trial results. Researchers can apply with a proposal, which will be reviewed by an access committee. If the request is approved, an anonymised set of data will be provided on the condition of a signed data access agreement.

## Research in context

### Evidence before the study

Rani and colleagues conducted a recent review of the available evidence for oral rehydration solution (ORS) therapy for children (aged 0–5 years) with severe acute malnutrition complicated by acute diarrhoea and dehydration up to December, 2022, using the search terms “malnutrition” AND “children” AND “rehydration” AND “oral” AND ReSoMaL on the following search engines: PubMed, MEDLINE, Scopus, Cochrane Database of Systematic Review, ClinicalTrials.gov, Google Scholar, and Google. Studies involving children with chronic or persistent diarrhoea lasting 14 days or longer were excluded. They identified seven randomised controlled trials involving 964 children, all published in English, conducted in low-resource settings. Six studies were conducted in Asia (Bangladesh [n=2], India [n=3], and South Korea [n=1]), and one was conducted in Africa (Egypt [n=1]). The trials compared various ORS therapies, including low-sodium rehydration solution for malnutrition (ReSoMal), standard low-osmolarity WHO-ORS, and old WHO-ORS. Both trials evaluating ReSoMal (*vs* either low-osmolarity WHO-ORS [Memon and colleagues, 2020] or old WHO-ORS [Alam and colleagues, 2003]) showed similar efficacy. Both studies reported significantly more episodes of hyponatremia by 24 h in the ReSoMal group than in the old WHO-ORS or standard WHO-ORS groups. Severe hyponatremia resulted in three children experiencing convulsions. Alam and colleagues (2003) showed that ReSoMaL corrected basal hypokalaemia in a greater proportion of children by 24 h (36% *vs* 5%, p=0·0006) and by 48 h (46% *vs* 16%, p=0·004) compared with old WHO-ORS. No additional randomised trials have been published since this review (search date Nov 8, 2025). Since no trial has been conducted in sub-Saharan Africa, this remains a key knowledge gap.

### Added value of this research

GASTROSAM was a stratified trial that enrolled 415 children admitted to hospital with severe acute malnutrition across four African countries. Children with severe (stratum A) and moderate (stratum B) dehydration were randomly assigned to receive ReSoMal or WHO-ORS. For comparison of the choice of ORS for ongoing rehydration, the primary outcome, change in sodium concentration at 24 h after baseline, did not differ between the ReSoMaL or standard WHO-ORS groups. At 8 h, severe hyponatraemia was significantly more common in the ReSoMal group than in the WHO-ORS group and hypernatraemia at 8 h and 24 h was rare. Hypokalaemia rates at 8 h and 24 h were similar between the groups. Mortality rates were similar between study groups and within each stratum. Both strategies were considered to be safe as we found no evidence of fluid overload, including pulmonary oedema or cardiac overload.

### Implications of all the available evidence

Standard low-osmolarity WHO-ORS, recommended for non-malnourished children, is a safe option for rehydration in children with severe malnutrition with moderate to severe dehydration due to gastroenteritis. Three studies have now shown that ReSoMal was associated with a significantly higher frequency of severe hyponatraemia and in one study this was associated with life-threatening consequences. Current recommendations for the type of ORS should be updated to remove the distinction between children with severe acute malnutrition and non-severe acute malnutrition.
