# Supplementary material for: Oral rehydration for severe malnutrition in children with moderate and severe dehydration (GASTROSAM): a phase 2, open-label, superiority randomised controlled trial
Source: Lancet Child Adolesc Health. Author manuscript; Available in PMC 2026 Sep 29. (PMC7619504; doi:10.1016/S2352-4642(25)00371-2)
Supplement: Supplementary file 1 [file EMS216163-supplement-Appendix_1.pdf]

THE LANCET
Child & Adolescent Health

Supplementary appendix 1

This appendix formed part of the original submission and has been peer reviewed. We post it as supplied by the authors.

Supplement to: Maitland K, Sainna H, Connon R, et al. Oral rehydration for severe malnutrition in children with moderate and severe dehydration (GASTROSAM): a phase 2, open-label, superiority randomised controlled trial. *Lancet Child Adolesc Health* 2026; published online March 10. https://doi.org/10.1016/S2352-4642(25)00371-2.

Supplement

Contents

List of Investigators and Committee Members 2
List of Investigators 2
List of Committee Members 3
Methods 4
Trial Sites 4
Trial Sites expertise and training 5
(a) Trial Design: Hypothesis and Objective 5
(b) Further Details of Allocation 6
(c) Further Details of Endpoint Ascertainment 6
(d) Further Details of justification for sample size calculation and overall enrolment numbers 7
(e) Protocol Versions 8
(f) Ethics approvals 9
(g) Interim Analyses 10
(h) Oral rehydration solutions: standard WHO-ORS manufacturers and content 11
Table S1 Baseline characteristics for those in Stratum A (severe dehydration) 12
Table S2: Baseline characteristics for those in Stratum B (moderate dehydration) 14
Table S3 Time to start ORS (from randomisation) 16
Table S4: Adherence to ORS rehydration strategy, Stratum A only 16
Table S5: Adherence to ORS rehydration strategy, Stratum B only 17
Table S6 Completeness of follow up 17
Tables S7 Outcomes Stratum A: severe dehydration 18
Tables S8 Outcomes Stratum B: Moderate dehydration 20
Table S9 Post-Hoc Analysis: mortality at day 3 and day 7 22
Table S10. Pre-specified sub-group analyses for the primary outcome of change in sodium at 24 hours 22
Figure S1 Trial Flow 23
References 24
Trial Protocol 25
Trial Statistical Analysis Plan 26

List of Investigators and Committee Members

List of Investigators

Name	Position	Affiliation	Country
Phyles Maitha	Trial administrator	Kilifi Clinical Trials Facility KEMRI Wellcome Trust Research Programme, Kilifi	Kenya
Nchafatso Obonyo	Paediatrician	KEMRI Wellcome Trust Research Programme, Kilifi	Kenya
Jay Berkley	Paediatrician	KEMRI Wellcome Trust Research Programme, Kilifi	Kenya
Kirsty Houston	Paediatrician	KEMRI Wellcome Trust Research Programme, Kilifi	Kenya
Victor Bandika	Consultant Paediatrian/ (Site-PI)	Coast General Teaching and Referral Hospital	Kenya
Laura Mwalekwa	Clinical Officer	Coast General Teaching and Referral Hospital	Kenya
George Passi	Medical Officer	Mbale Regional Referral Hospital	Uganda
Moses Oluput-Olupot	Study coordinator	Mbale Regional Referral Hospital	Uganda
Charles B Okalebo	Pharmacist	Mbale Regional Referral Hospital	Uganda
Rita Muhindo	Head of laboratory	Mbale Regional Referral Hospital	Uganda
Grace Abongo	Data Manager	Mbale Regional Referral Hospital	Uganda
Margret Nakuya	Medical Officer	Soroti Regional Referral Hospital	Uganda
Hastings C Onu	Epidemiologist	Médecins Sans Frontières (MSF), Abuja	Nigeria
Mohammed Ashir Garba	Clinician	Faculty of Medicine, University of Maiduguri Teaching Hospital, Maiduguri	
Mark Lee	Paediatric Advisor	Médecins Sans Frontières, Geneva	Switzerland
Grace Mambula	Pharmacovigilance Specialist	Epicentre, Paris,	France
Johanna Thomson	Paediatric Working Group Lead	Médecins Sans Frontières, Paris	France

List of Committee Members

Trial Steering Committee

Professor Elizabeth Molyneux, OBE (Chairman): College of Medicine, Blantyre, Malawi

Dr Jane Crawley University of Oxford, United Kingdom

Dr Irene Lubega Department of Paediatrics Makerere University College of Health Sciences, Uganda

Professor William Macharia Department of Paediatrics and Child Health, Aga Khan Hospital, Nairobi, Kenya.

Independent Data Monitoring Committee

Prof. Timothy Peto (Chair) University of Oxford, Oxford, United Kingdom

Dr. Jennifer Thompson (statistician) (for 3 reviews), London School of Hygiene and Tropical Medicine, London, United Kingdom

Dr Nuredin Mohammed (statistician) (for 1 review), MRC Unit at The Gambia, London School of Hygiene and Tropical Medicine, The Gambia.

Professor Dr. Edison Mworozi Department of Paediatrics, Mulago Hospital, Kampala, Uganda

Professor Philippa Musoke Makerere University College of Health Sciences, Kampala, Uganda.

Methods

Trial Sites

Kilifi County Hospital, Kilifi, Kenya
Annual rates of pediatric and neonatal admission to KCH are 4500. KCH has a general pediatric ward (staffed by county clinical staff) and a 5-bedded high dependency unit which frequently has a 200% bed occupancy (staffed by dedicated research teams) and a neonatal side ward with capacity for 9 incubators/cots.

Coast General Hospital, Mombasa, Kenya
This is a government hospital with a general pediatric ward (staffed by county clinical staff) and has no dedicated high dependency care unit.

Mbale Regional Referral Hospital, Mbale, Uganda
Annual rates of pediatric and neonatal admission to MRRH are 19000. MRRH has a general pediatric ward (staffed by county clinical staff) and has no dedicated high dependency care unit.

Soroti Regional Referral Hospital, Soroti, Uganda
Annual rates of pediatric and neonatal admission to SRRH are 8500. SRRH has a general pediatric ward (staffed by county clinical staff) it has no dedicated high dependency care unit.

Magaria District Hospital, Magaria, Niger
Magaria District Hospital is a secondary-level hospital managed by the Ministry of Public Health. It serves a largely rural catchment area (population 931 254) in south-central Niger, and its pediatric and nutrition wards are supported by Médecins Sans Frontières. In 2024, 10722 children <5 years were admitted as inpatients in the pediatrics department, 11306 to inpatient therapeutic feeding centers, and 28012 to malaria-specific units during the seasonal malaria peak.

Nilefa Kiji MSF Hospital, Maiduguri, Nigeria

Nilefa Keji Hospital, operated by Médecins Sans Frontières Operational Centre Brussels (MSF-OCB), in Borno State, Nigeria (population ~6.11 million). It provides care to children with acute malnutrition. In 2024, the Inpatient Therapeutic Feeding Centre (ITFC) admitted 16,280 patients, with 12.4% requiring ICU care, with average Bed Occupancy Rate (BOR) of 87% but reached 167% during peak season (August to October). The mortality rate was approximately 4%. The ITFC has an Emergency Room (ER), Intensive Care Unit (ICU) of 20 beds (expanded to 40 during highest peak of August-October), phase I and transition phase beds (total 120 bed) and an isolation unit (20 beds, up to 38 beds during peak). Overall bed capacity varies seasonally from 200 to 400 beds. Staffing ranges from 400 in the low season to 800 in peak periods. The GASTROSAM trial ward was a nine-bed unit in the ER Extension that had dedicated staff of 4 doctors, 8 nurses, 4 nutrition assistants, a counsellor, and a health promoter.

Trial Sites expertise and training

The study was conducted by dedicate trial teams specifically employed to run the trial. At each centre there was a specialist paediatrician overseeing the team. For the Niger and Nigerian site operational specialists from Medicine Sans Frontieres worked closely with the trial management team based in Kenya. The Kenyan and Ugandan sites had experienced researchers who have previously run large Phase III trials. Before the start of participant recruitment all staff we trained on clinical sign recognition, clinical management and specially how to recognize fluid overload. Each member of the team had accesses the 50-page manual of operations which detailed every aspect of the trial. Weekly trial management meetings were held between the local trial sites and trial management team. Monthly meetings involved international investigators.

(a) Trial Design: Hypothesis and Objective

We had two hypothesis which relate to i) immediate management of severe dehydration and ii) Type of oral rehydration solution to use in both severe dehydration and moderate dehydration

For children with SAM with severe dehydration we hypothesized that standard intravenous regime WHO PlanC (100mls/kg over 3-5 hours) used for non-SAM gastroenteritis with severe dehydration will result in betteroutcomes than the current very conservative SAM rehydration recommendations. In addition, we propose that the rate of rehydration may be critical and hypothesize that 100mls/kg over 8 hours in SAM children will result in fewer fluid related adverse effects than rapid WHO Plan C guideline.

We also propose that standard oral rehydration solutions (ORS) may be equally as effective with fewer side effects than low-sodium Rehydration Solution for Malnutrition (ReSoMal).

Major Objectives:

To compare the rate and volume of rehydration in children with signs of severe dehydration (see study population) secondary to gastroenteritis on a primary endpoint of 96-hour mortality:

(i) Current standard WHO rehydration protocol Plan C usually used in non-SAM children including boluses for shock (rapid rehydration)
(ii) A slower rehydration regimen using the same total volume (100ml/kg) over 8 hours, irrespective of age and no boluses for shock (slow rehydration)
(iii) The current WHO restrictive intravenous rehydration strategy for SAM children (control).

In children with diarrhea complicated by moderate or 'some' dehydration (see study population) and as follow-on rehydration post-intravenous rehydration in those with severe dehydration whether oral rehydration with

(i) WHO standard oral rehydration solution (ORS) for non-SAM[21] is safer and results in less hyponatremia and better outcomes compared to
(ii) current recommendation advocating low sodium (ReSoMal) ORS[4]

Stratum A randomisation R1

In stratum A eligible children with severe dehydration were randomly assigned 1:1:2 i) liberal: rapid rehydration (following WHO Plan C : 100 ml/kg Ringer's Lactate over 3-6 hours according to age with boluses (20 ml/kg) for those with shock) ii) liberal: slow rehydration (100 ml/kg Ringer's Lactate over 8h and no boluses for shock) or iii) control: oral rehydration solution 5ml/kg every 30 minutes for the first 2hours followed by 5–10 ml/kg per hour for the next 4–10h alternating hourly with F75 milk formula, with boluses of Ringer's Lactate (15 ml/kg) for those with shock). Shock was defined as the presence of all of the following: cold peripheries (meaning hands and-or feet), a weak and fast pulse (rate not specified) and a capillary refilling time >3s[15] . The oral rehydration strategy for the control arm was determined by the second factorial randomisation (R2). See Trial flow (**Figure S1) .**

(b) Further Details of Allocation

Randomisation lists were generated (by RC) separately for each stratum and kept at the MRC CTU at UCL, London. Opaque sealed envelopes containing the randomized allocation(s) were prepared at the Clinical trials facility, KWTRP, Kilifi and shipped to the clinical trial sites. The envelope contained the actual allocation for intravenous and oral strategies which was visible only once opened. The cards were numbered consecutively within each stratum and opened in numerical order. This method was used to ensure that acutely unwell children could be treated immediately at the emergency ward, without having to rely on a computer or the internet to obtain a randomized allocation.

The allocation ratio was 2:1:1 so that we could combine the two liberal strategies together and for this total to match those in the standard of care arm. This would ensure the power for this comparison, given expected mortality rates, balanced against what was realistic for recruitment in these settings.

(c) Further Details of Endpoint Ascertainment

SAEs were defined following the International Committee for Harmonisation as events which led to death, were life-threatening, caused or prolonged hospitalization (excluding elective procedures), caused permanent disability, or were other medical conditions or with a real, not hypothetical risk of one of the previous categories.

At each clinical review the clinicians were asked to observe for solicited SAEs which included shock and clinical signs of fluid overload. Severe adverse events used the standardized definitions and internal review processes of these events will be carried out using the same criteria as were used in the FEAST trial[1]. All relevant adverse events are reported in the case report form and SAE form. The reporting procedure was captured within the safety reporting SOP.

(d) Further Details of justification for sample size calculation and overall enrolment numbers

Justification for endpoint and sample size calculation

Children with severe malnutrition and dehydration secondary to diarrhoea have a high frequency of hyponatraemia[2]. Since RESOMAL contains much less sodium than standard ORS our major concern was to prevent the development or prolong the exposure to severe hyponatraemia (defined as sodium < 125mmol/l) since this is an independent risk factor for early mortality[2]. The data available prior to the development of the trial protocol, reported in Section 14 (Sample size determination), was very sparse with none being available from studies conducted in African children.

Two small studies in Asia, both of which excluded children with severe electrolyte derangement, compared ORS versus ReSoMal[3,4].

One compared WHO ORS group (n=64) versus RESOMAL (n=64). Sodium levels were similar at baseline (median 132mols/l) but was significantly lower at 24 hours by - 2.8(-4.9, to -0.7)p=0.01 in ReSoMal group[3]. One child (2%) developed severe hyponatraemia (sodium ≤120mmol/l) in the ORS arm versus 3/65 (5%) in the ReSoMal arm. In the ResoMAL arm one child developed seizures secondary to hyponatraemia.

A second trial comparing ReSoMal (n=55) vs. hypo- osmolar ORS (n=55) where median baseline sodium levels were 141 mmol/l showed a greater proportion of children receiving ReSoMal developed hyponatraemia defined as sodium < 130 mmols/L) (15.4% vs. 1.9%, p=0.03)[4].

Given the low frequency of the development of severe hyponatraemia reported in these trials, and given that GASTROSAM was a Phase II trial we opted for a continuous variable (sodium) for the endpoint. Thus, primary endpoint for oral rehydration strategies was change in sodium at 24 hours. We estimated, from the available data[3], would give >90% power to detect a 3mmol/l difference (higher) in sodium levels (assuming mean of 132mmol/l (SD= 7) at 24 hours between the ORS verses ReSoMal (total n=336, 168 per arm (32 some/moderate dehydration, 136 severe dehydration).

Final Enrolment numbers

We had anticipated enrolling only 64 children in the moderate dehydration arm, however when Medicines Sans Frontieres joined the trial, the initial enrolment was just in advance of their peak season and most cases had moderate dehydration and we agreed to not stop recruitment after 64 had been enrolled. This was reported to the local ethics committees who raised no major concerns. The independent Data Monitoring Committee were also informed. They had no safety concerns in their interim analysis. The final overall numbers enrolled were 143 in the moderate dehydration arm, an important addition to the data available in Africa in this group for oral rehydration since there were none previously. Specifically on the safety of ORS in this subgroup since no fluid overload events were reported.

(e) Protocol Versions

Version 1 to Version 1.2
Version 1.0 was the original protocol submitted for ethical approval to Imperial College Research Ethics Committee in February 2018 and approved (20th March) following additional background information included in the Protocol (version 1.1: 5th June 2019). Further clarifications were requested following review by Mbale Regional Referral Hospital Research Ethics, Uganda Version 1.2 was approved by MRRH-REC on June 14th 2019.
Version 1.2 to Version 2.0
Minor Amendment- additional staff members added to replace departing staff (Sept 23rd 2019) prior to any enrollment.
Version 2.0 to 2.2
Major changes:

1) Dropping of the Mulago site, Kampala, Uganda
2) Addition of a second site in Kenya, Coast General Hospital, Mombasa

Minor changes

1) Changes in members of the investigator teams (removal of Mulago site investigators and addition of new investigators for Kilifi and Mombasa sites; change of Imperial administrator)

Version 3.0 (dated 25th November 2022)
Major Changes

1) Addition of an extra study sites in Niger and Nigeria and addition of the 2 new study co-investigators
   Owing to slow enrollment in both Uganda and Kenya, Medicines Sans Frontières (MSF) were approached to join the trial. Three MSF centres were selected, one in Nigeria and two in Niger (with three additional principal investigators for these sites). It was anticipated that the main site would be Magaria with a reserve site in case of slow enrollment. The decision to involve MSF, who have a substantial experience in managing children with severe malnutrition and have large case numbers, was endorsed by the independent Trial Steering Committee (TSC) and subsequently approved by the funders (MRC UKRI). Pragmatically we agreed that 2-3 patient per day would be enrolled at these sites in order to ensure quality of trial conduct.
2) Change of primary endpoint for intravenous rehydration stratum and increase in sample size
   A concern raised by the investigators and TSC was whether, if the trial were to show significantly better survival for those receiving liberal rehydration, this would be sufficient to advocate for guideline change given that mortality was a secondary endpoint in protocol Version 2.1, and urinary output at 8 hours was the primary endpoint. The primary endpoint was therefore changed to mortality at 96 hours post randomisation; and a sample size to reflect this new endpoint was calculated.
3) The change in primary endpoint and modest increase in sample size was approved by the TSC and the funders (MRC UKRI).

Minor Changes

4) Clarification of follow up <u>at</u> Day 7 and Day 28 (post enrollment/randomisation) rather than post discharge as incorrectly stated in the study protocol. There were inconsistencies in the protocol where Day 7 and Day 28 (post enrollment) were indicated on the Tables representing study

procedures but in the text this was written as Day 7 and Day 28 (post discharge). The two secondary endpoints that this would affect were weight and MUAC at Day 7. For patients already enrolled in the trial we reviewed the data gathered in the trial to date and were reassured that these outcomes could be obtained from the daily weight and MUAC measures for most where they were discharged after 7 days.

5) Clarification that sodium levels at 24 hours and 8 hours were post-randomisation rather than completion of iv treatment (post-iv) (Summary and Page 20)
6) Addition of Day 3 anthropometry to record post rehydration weight/MUAC since some children were discharged before Day 7 (Summary, Table and Page 21)
7) Simplification of additional study procedures, especially relating to urine collection and sample processing where facilities are not available.
8) Many additional procedures focused on providing data for physiological measures (eg plasma and urine osmolarity, endotoxin and other biomarkers) were indicated as optional and only where laboratory facilities permit.
9) All safety endpoints were to be monitored as per the original protocol.

GASTROSAM SAP v4.0 reflected protocol v3.0 however the addition of weight and MUAC change at day 3 was inadvertently not added. In addition, electrolyte measurement at 24 hours was mentioned in the protocol but change in potassium was not explicitly listed in the SAP (change in sodium was already listed at a primary endpoint). It was added as an exploratory outcome as ReSoMal (despite being introduced to avert sodium overload) has been additionally championed as it provides a superior potassium correction due to its high potassium content, so the investigators wanted to report the data collected. The Trial Steering Committee and Data Monitoring Committee approved the addition of these outcomes to the reporting of the trial.

(f) Ethics approvals

Imperial College Research Ethics Committee
Medical School Building
St Marys Campus London
W2 1PG
UK
Protocol Number 18IC4427
Date 10/04/2018

Mbale Regional Hospital Research and Ethics Committee
Pallisa Road
Mbale
PO Box 921
Uganda
Protocol Number MRRH-REC OUT 107/2018
Date 14/06/2019

KEMRI Scientific and Ethics Committee
Mbagathi Road
POB584840
Nairobi
Kenya
Protocol Number KEMR/SERU/CGMR-C/204/4072
Date 25/08/2020

(g) Interim Analyses

The DMC Charter outlined the responsibilities of the DMC and stated: The DMC should inform the Chair of the TSC if, in their view, the results are likely to convince a broad range of clinicians, including those supporting the trial and the general clinical community, that, on balance, one trial arm is clearly indicated or contraindicated for all participants or a particular category of participants. It is important that the implications (e.g. ethical, statistical, practical, and financial) for the trial be considered before any recommendation is made.
The statistical implications would be based on the Haybittle-Peto rule, even though not explicitly stated, as this is used in practice by trials run by this group with the same DMC. As this rule was implicitly being used then the number of interim analyses was not fixed in advance, as it has a trivial impact on the final alpha/Type I error.

The DMC met four times. The trial statisticians prepared a confidential report including interim analyses as outlined in the statistical analysis plan (page 12) that was circulated only to the DMC committee members. The meeting dates and the DMC conclusions are given below. These conclusions were reported to the TSC and trial investigators.

11th June 2020:

We have no safety concerns and see no reason for any of the trial arms to stop, or be modified in any way.
We appreciated the detail given in the open session regarding the rational and progress of the trial including challenges in recruitment. We understand the difficulties, and note the efforts that were made to increase recruitment prior to the trial being paused due to COVID19.

12th December 2022:

We have no safety concerns and see no reason for any of the trial arms to stop, or be modified in any way.
We appreciated the detail given in the open session regarding the rational and progress of the trial including details of the proposed changes. We are pleased that the trial is expanding to new sites to ensure continued recruitment.

23rd May 2024:

We have no safety concerns and see no reason for any of the trial arms to stop, or be modified in any way.

We are pleased that MSF has supported this trial and that there is an increased number of sites open. We note high levels of follow up and hope for continuation of current good levels of recruitment.

5th September 2024:

We have no safety concerns and see no reason for any of the trial arms to stop or be modified in any way.
We congratulate the site teams on their current recruitment levels and good retention rates.

(h) Oral rehydration solutions: standard WHO-ORS manufacturers and content

Niger site
Oral Rehydrations Salts 20,5g- Low osmolarity
KBI Pharmaceuticals GmbH & Co. KG
Eichborndamm 131-165
Berlin

Nigeria Site
Oral rehydration Salts BP
FDC Limited
India

Kenya Sites
Prolyte Oral Rehydration Salts
Cipla
Elys Chemical Industries Ltd
Road B, Off Enterprise Way
Nairobi

Uganda Sites
Oralyte,
Medipharm East Africa Ltd
Kampala
Uganda

Each sachet of ReSoMal contains 42 g powder for solution to be diluted in 1 litre of purified/boiled and cooled water (containing glucose 125 mmol/l; sodium 45 mmol/l, potassium 40 mmol/l, chloride 70mmol/l, citrate 7mmol/l, magnesium 3 mmol/l, zinc 0.3 mmol/l copper 0.045 mmol/l. The osmolarity of the prepared solution is 300 mmol/l). The WHO-ORS sachet contains 20.5 g of powder for dilution in 1 litre of boiled and cooled water and contains glucose 75 mmol/l, sodium 75mmol/l, chloride 65 mmol/l and citrate 10mmol/l. The osmolarity of the prepared solution is 245 mmol/l.

Table S1 Baseline characteristics for those in Stratum A (severe dehydration)

	ReSoMal (N=137)	WHO-ORS (N=135)	Total (N=272)
Site: Mbale, Uganda	4 (3%)	3 (2%)	7 (3%)
Soroti, Uganda	2 (1%)	0 (0%)	2 (1%)
Kilifi, Kenya	5 (4%)	4 (3%)	9 (3%)
Coast, Kenya	1 (1%)	1 (1%)	2 (1%)
Maiduguri, Nigeria	56 (41%)	57 (42%)	113 (42%)
Magaria, Niger	69 (50%)	70 (52%)	139 (51%)
Sex - male	60 (44%)	66 (49%)	126 (46%)
Female	77 (56%)	69 (51%)	146 (54%)
Age months	14 (9, 24)	12 (9, 21)	13 (9, 23)
Weight (kg)	5.5 (4.7, 6.2)	5.1 (4.5, 6.0)	5.2 (4.6, 6.1)
Weight-for-height z-score	-4.7 (-5.5, -4.1)	-4.9 (-5.8, -4.3)	-4.8 (-5.6, -4.2)
MUAC (cm)	10.5 (9.6, 11.0) [N=136]	10.0 (9.5, 11.0) [N=135]	10.3 (9.5, 11.0) [N=271]
Kwashiorkor phenotype	6 (4%)	5 (4%)	11 (4%)
Altered consciousness[~]	53 (39%)	51 (38%)	104 (38%)
Restless or irritable	40 (29%)	46 (34%)	86 (32%)
Sunken eyes	133 (97%)	134 (99%)	267 (98%)
Abdominal Skin pinch 1-2sec	10 (97%)	15 (11%)	25 (9%)
Abdominal Skin pinch >2sec	125 (91%)	117 (87%)	242 (89%)
Unable to take or retain oral fluids	107 (78%)	108 (80%)	215 (79%)
Fever (temperature >37.5°C)	52/137 (38%)	42/134 (31%)	94/271 (35%)
Severe Tachypnoea (>40 breaths/minute)	34 (25%)	35 (26%)	69 (25%)
Oxygen saturation (%)	100 (98, 100) [N=137]	100 (99, 100) [N=134]	100 (98, 100) [N=271]
Heart rate (beats/minute)	135 (116, 152)	138 (120, 156)	137 (118, 154)
Severe Tachycardia[#]	65 (47%)	66 (49%)	131 (48%)
Capillary refill time >3seconds	17 (12%)	23 (17%)	40 (15%)
Weak pulse	24 (18%)	27 (20%)	51 (19%)
Cold peripheries	22/136 (16%)	19/135 (14%)	41/271 (15%)
Moderate hypotension	42/131 (32%)	34/130 (26%)	76/261 (29%)
WHO Shock	12 (9%)	13 (10%)	25 (9%)
Indrawing	14/137 (10%)	15/133 (11%)	29/270 (11%)
Deep breathing	12/137 (9%)	14/133 (11%)	26/270 (10%)
Lung crepitations	19/134 (14%)	14/129 (11%)	33/263 (13%)
Bloody diarrhoea	27/137 (20%)	27/132 (20%)	54/269 (20%)
History of vomiting	106/137 (77%)	109/133 (82%)	215/270 (80%)
Appetite (1-10 scale)	3 (2, 5) [N=136]	3 (2, 5) [N=134]	3 (2, 5) [N=270]
Still breastfeeding	67/134 (50%)	71/133 (53%)	138/267 (52%)
Currently on nutritional feed	12/129 (9%)	16/134 (12%)	28/263 (11%)
Taken Oral Rehydration Solution in this illness	84/137 (61%)	75/134 (56%)	159/271 (59%)
Admitted to another facility in this illness	74/130 (57%)	67/134 (50%)	141/264 (53%)

	ReSoMal (N=137)	WHO-ORS (N=135)	Total (N=272)
Diarrhoea in last 6 months	72/135 (53%)	73/134 (54%)	145/269 (54%)
Previous admission for malnutrition	39/129 (30%)	40/134 (30%)	79/263 (30%)
**Laboratory measures**			
Hypoglycaemia (glucose <3 mmol/l)	12/134 (9%)	13/132 (10%)	25/266 (9%)
Sodium (mmol/l)	124 (118, 129) [N=132]	124 (119, 129) [N=130]	124 (118, 129) [N=262]
Severe hyponatraemia (sodium <125 mmol/l)	70/132 (53%)	67/130 (52%)	137/262 (52%)
Potassium (mmol/l)	2.7 (2.0, 3.4) [N=129]	2.6 (2.0, 3.3) [N=129]	2.7 (2.0, 3.3) [N=258]
Severe hypokalaemia (potassium <2.5 mmol/l)	59/129 (46%)	56/129 (43%)	115/258 (45%)
Chloride (mmol/l)	97 (93, 104) [N=129]	99 (92, 105) [N=130]	98 (92, 105) [N=259]
Bicarbonate (mmol/l)	14 (11, 19) [N=58]	14 (10, 18) [N=55]	14 (10, 19) [N=113]
Children living with HIV	0 (0%)	2 (1%)	2 (1%)
Malaria rapid test positive	24 (18%)	21 (16%)	45 (17%)
Haemoglobin (g/dL)	11.6 (10.1, 12.7) [N=131]	11.2 (9.6, 12.6) [N=127]	11.4 (9.7, 12.6) [N=258]
Severe anaemia (hb < 5 g/dL)	0/131 (0%)	0/127 (0%)	0/258 (0%)
Blood culture positive	4/49 (8%)	8/49 (16%)	12/98 (12%)

~Altered consciousness: : The level of consciousness was assessed on the basis of the four-component AVPU scale, with “Alert” indicating that the patient is aware and can respond independently, “Verbal” indicating that the patient responds only to verbal stimuli, “Pain” indicating that the patient responds only to a painful stimulus, and “Unresponsive” indicating that the patient does not respond to verbal or painful stimuli. Altered level of consciousness was considered to be a status of Verbal, Pain, or Unresponsive.
[#]Severe Tachycardia defined as a heart rate > 160 bpm in children aged <12 months; >120 bpm if aged ≥12 months.

Table S2: Baseline characteristics for those in Stratum B (moderate dehydration)

	ReSoMal (N=69)	WHO-ORS (N=74)	Total (N=143)
Site: Mbale, Uganda	6 (9%)	7 (9%)	13 (9%)
Soroti, Uganda	8 (12%)	8 (11%)	16 (11%)
Kilifi, Kenya	7 (10%)	8 (11%)	15 (10%)
Coast, Kenya	0 (0%)	3 (4%)	3 (2%)
Maiduguri, Nigeria	24 (35%)	24 (32%)	48 (34%)
Magaria, Niger	24 (35%)	24 (32%)	48 (34%)
Sex - male	47 (68%)	45 (61%)	92 (64%)
Female	22 (32%)	29 (39%)	51 (36%)
Age months	15 (10, 24)	12 (10, 20)	14 (10, 23)
Weight (kg)	5.9 (5.3, 7.4)	5.7 (4.8, 6.5)	5.8 (5.0, 6.8)
Weight-for-height z-score	-4.3 (-5.1, -3.6)	-4.5 (-5.4, -3.8)	-4.4 (-5.3, -3.7)
MUAC (cm)	11.0 (10.0, 11.5) [N=68]	10.5 (9.5, 11.1) [N=74]	10.5 (9.8, 11.4) [N=142]
Kwashiorkor phenotype	5 (7%)	8 (11%)	13 (9%)
Altered consciousness~	0 (0%)	0 (0%)	0 (0%)
Restless or irritable	42 (61%)	44 (59%)	86 (60%)
Sunken eyes	67 (97%)	72 (97%)	139 (97%)
Abdominal Skin pinch 1-2sec	62 (90%)	64 (86%)	126 (88%)
Abdominal Skin pinch >2sec	1 (1%)	1 (1%)	2 (1%)
Unable to take or retain oral fluids	2 (3%)	2 (3%)	4 (3%)
Fever (temperature >37.5°C)	23 (33%)	26 (35%)	49 (34%)
Severe Tachypnoea (>40 breaths/minute)	25 (36%)	22 (30%)	47 (33%)
Oxygen saturation (%)	100 (98, 100)	100 (98, 100)	100 (98, 100)
Heart rate (beats/minute)	139 (128, 161)	138 (122, 157)	138 (126, 160)
Severe Tachycardia#	50 (72%)	41 (55%)	91 (64%)
Capillary refill time >3seconds	0 (0%)	0 (0%)	0 (0%)
Weak pulse	2 (3%)	2 (3%)	4 (3%)
Cold peripheries	2 (3%)	4 (5%)	6 (4%)
Moderate hypotension	9/67 (13%)	8/73 (11%)	17/140 (12%)
WHO Shock	0 (0%)	0 (0%)	0 (0%)
Indrawing	2 (3%)	4 (5%)	6 (4%)
Deep breathing	1 (1%)	6 (8%)	7 (5%)
Lung crepitations	5/68 (7%)	10/73 (14%)	15/141 (11%)
Bloody diarrhoea	3 (4%)	13 (18%)	16 (11%)
History of vomiting	43 (62%)	55 (74%)	98 (69%)
Appetite (1-10 scale)	4 (2, 5) [N=69]	3 (2, 5) [N=72]	3 (2, 5) [N=141]
Still breastfeeding	28/69 (41%)	38/73 (52%)	66/142 (46%)
Currently on nutritional feed	6/66 (9%)	7/74 (9%)	13/140 (9%)
Taken Oral Rehydration Solution in this illness	26 (38%)	37 (50%)	63 (44%)
Admitted to another facility in this illness	28/66 (42%)	30/74 (41%)	58/140 (41%)
Diarrhoea in last 6 months	23 (33%)	34 (46%)	57 (40%)
Previous admission for malnutrition	13/67 (19%)	21/73 (29%)	34/140 (24%)

	ReSoMal (N=69)	WHO-ORS (N=74)	Total (N=143)
**Laboratory measures**			
Hypoglycaemia (glucose <3 mmol/l)	5/68 (7%)	6/74 (8%)	11/142 (8%)
Sodium (mmol/l)	128 (124, 133) [N=68]	129 (124, 132) [N=71]	129 (124, 132) [N=139]
Severe hyponatraemia (sodium <125 mmol/l)	24/68 (35%)	21/71 (30%)	45/139 (32%)
Potassium (mmol/l)	3.0 (2.5, 3.7) [N=68]	2.9 (2.3, 3.8) [N=71]	3.0 (2.4, 3.7) [N=139]
Severe hypokalaemia (potassium <2.5 mmol/l)	16/68 (24%)	22/71 (31%)	38/139 (27%)
Chloride (mmol/l)	101 (95, 105) [N=63]	100 (97, 104) [N=65]	100 (96, 104) [N=128]
Bicarbonate (mmol/l)	14 (11, 16) [N=25]	14 (12, 18) [N=28]	14 (11, 17) [N=53]
Children living with HIV	5 (7%)	4 (5%)	9 (6%)
Malaria rapid test positive	16 (23%)	10 (14%)	26 (18%)
Haemoglobin (g/dL)	10.5 (8.9, 12.1) [N=67]	10.0 (9.1, 11.9) [N=72]	10.1 (9.1, 12.1) [N=139]
Severe anaemia (hb < 5 g/dL)	1/67 (1%)	2/72 (3%)	3/139 (2%)
Blood culture positive	5/35 (14%)	3/40 (8%)	8/75 (11%)

~Altered consciousness: : The level of consciousness was assessed on the basis of the four-component AVPU scale, with “Alert” indicating that the patient is aware and can respond independently, “Verbal” indicating that the patient responds only to verbal stimuli, “Pain” indicating that the patient responds only to a painful stimulus, and “Unresponsive” indicating that the patient does not respond to verbal or painful stimuli. Altered level of consciousness was considered to be a status of Verbal, Pain, or Unresponsive.

#Severe Tachycardia defined as a heart rate > 160 bpm in children aged <12 months; >120 bpm if aged ≥12 months.

Table S3 Time to start ORS (from randomisation)

	ReSoMal	WHO-ORS	Total
**Strata A & B**	0.5 (0.2-4.2)	0.6 (0.2-4.3)	0.5 (0.2-4.3)
**Strata A**			
Overall	1.6 (0.3-7.8)	0.9 (0.3-7.2)	1.4 (0.3-7.7)
Control: ORS	0.3 (0.2-0.5)	0.3 (0.1-0.6)	0.3 (0.2-0.5)
Liberal: Rapid	5.7 (4.0-7.1)	5.2 (4.0-6.8)	5.3 (4.0-6.9)
Liberal: Slow	8.7 (8.4-9.2)	8.6 (8.4-9.3)	8.7 (8.4-9.2)
**Strata B**	0.3 (0.2-0.6)	0.3 (0.2-0.7)	0.3 (0.2-0.6)

Median (IQR) hours presented.

Table S4: Adherence to ORS rehydration strategy, Stratum A only

	ReSoMal	WHO-ORS	Total
**ReSoMal**			
Total	91 (66-122)	0 (0-0)	0 (0-91)
0-4 hours	30 (30-40)	0 (0-0)	0 (0-30)
4-8 hours	20 (10-20)	0 (0-0)	0 (0-20)
8-16 hours	25 (10-40)	0 (0-0)	0 (0-25)
**WHO ORS**			
Total	0 (0-0)	139 (89-195)	0 (0-137)
0-4 hours	0 (0-0)	75 (60-76)	0 (0-75)
4-8 hours	0 (0-0)	20 (0-40)	0 (0-20)
8-16 hours	0 (0-0)	29 (10-50)	0 (0-30)
**F75**			
N (%) received	129 (97%)	126 (95%)	255 (96%)
Total	114 (90-129)	116 (97-133)	115 (95-130)
Before starting ORS	0 (0-11)	0 (0-8)	0 (0-11)
0-4 hours	11 (10-19)	16 (0-17)	15 (0-17)
4-8 hours	20 (16-20)	17 (16-32)	20 (16-21)
8-16 hours	36 (30-49)	45 (32-49)	36 (32-49)

Median (IQR) mls/kg presented.
Times measured from start of oral rehydration. Total includes all oral intake recorded within 24 hours of starting any fluid (IV or ORS).

Table S5: Adherence to ORS rehydration strategy, Stratum B only

	ReSoMal	WHO-ORS	Total
**ReSoMal**			
Total	81 (70-110)	0 (0-0)	0 (0-80)
0-4 hours	30 (25-31)	0 (0-0)	0 (0-30)
4-8 hours	20 (10-20)	0 (0-0)	0 (0-20)
8-16 hours	20 (10-30)	0 (0-0)	0 (0-20)
**WHO ORS**			
Total	0 (0-0)	125 (96-152)	60 (0-126)
0-4 hours	0 (0-0)	75 (69-76)	24 (0-75)
4-8 hours	0 (0-0)	10 (0-26)	0 (0-11)
8-16 hours	0 (0-0)	18 (0-20)	0 (0-19)
**F75**			
N (%) received	69 (100%)	65 (88%)	134 (94%)
Total	114 (87-118)	114 (86-125)	114 (87-120)
0-4 hours	10 (10-15)	0 (0-16)	10 (0-16)
4-8 hours	20 (19-21)	16 (12-32)	20 (16-22)
8-16 hours	36 (32-43)	34 (32-49)	36 (32-49)

Median (IQR) mls/kg presented. Times measured from start of oral rehydration. Total includes all oral intake recorded within 24 hours of starting ORS.

Table S6 Completeness of follow up

	ReSoMal (N=206)	WHO-ORS (N=209)	Total (N=415)
**Visits considered complete:**			
7 days	201 (98%)	204 (98%)	405 (98%)
28 days	198 (96%)	194 (93%)	392 (94%)
**Child status at 7 days**			
Visit done	184 (89%)	189 (90%)	373 (90%)
Died	17 (8%)	15 (7%)	32 (8%)
Withdrawn	1 (0%)	0 (0%)	1 (0%)
Lost to follow up	0 (0%)	3 (1%)	3 (1%)
Missed visit	4 (2%)	2 (1%)	6 (1%)
**Child status at 28 days**			
Visit done	174 (84%)	174 (83%)	348 (84%)
Died	24 (12%)	20 (10%)	44 (11%)*
Withdrawn	1 (0%)	0 (0%)	1 (0%)
Lost to follow up	4 (2%)	6 (3%)	10 (2%)
Missed scheduled visit, had contact later	3 (1%)	9 (4%)	12 (3%)

Tables S7 Outcomes Stratum A: severe dehydration

	ReSoMal (N=137)	WHO-ORS (N=135)	Overall (N=272)	Estimate of effect* (Standard ORS vs ReSoMal) (95% CI)	p value
**Primary outcome (N with data)**	126	127	253		
Change in sodium (mmol/l) at 24 hours	6.9 (8.0)	5.8 (7.0)	6.3 (7.5)	-1.5 (-3.1, 0.1)	0.071
**Secondary outcomes**					
Mortality at 28 days	18 (13%)	13 (10%)	31 (11%)	0.74 (0.36, 1.52)†	0.42
Suspected pulmonary oedema - n (%)	0 (0%)	0 (0%)	0 (0%)		
Secondary heart failure - n (%)	0 (0%)	0 (0%)	0 (0%)		
Change in sodium level at 24 hours from 8 hours	2.9 (6.6)	0.6 (5.9)	1.7 (6.3)	-2.3 (-3.6, -0.9)	0.0013
**Biochemistry**					
**8 hours**					
Severe hyponatraemia - n (%)	48 (37%)	30 (24%)	78 (30%)	0.54 (0.31, 0.94)	0.030
Hypernatraemia - n (%)	3 (2%)	1 (1%)	4 (2%)	0.34 (0.03, 3.35)	0.33
Severe hypokalaemia - n (%)	50 (38%)	47 (38%)	97 (38%)	0.96 (0.58, 1.60)	0.89
Change in sodium (mmol/l) at 8 hours	4.2 (6.5)	5.2 (5.8)	4.7 (6.2)	0.9 (-0.5, 2.3)	0.23
Change in potassium at 8 hours	0.1 (0.9)	0.0 (0.9)	0.1 (0.9)	0.0 (-0.2, 0.2)	0.97
**24 hours**					
Severe hyponatraemia - n (%)	28 (22%)	28 (22%)	56 (22%)	1.00 (0.55, 1.81)	1.00
Hypernatraemia - n (%)	6 (5%)	3 (2%)	9 (4%)	0.49 (0.12, 2.00)	0.31
Severe hypokalaemia - n (%)	33 (26%)	29 (23%)	62 (25%)	0.83 (0.47, 1.48)	0.54
Change in potassium (mmol/l) at 24 hours	0.5 (0.9)	0.3 (1.0)	0.4 (0.9)	-0.1 (-0.3, 0.1)	0.23
**7 days**					
Severe hyponatraemia - n (%)	4 (4%)	4 (4%)	8 (4%)	0.89 (0.22, 3.66)	0.87
Hypernatraemia - n (%)	3 (3%)	0 (0%)	3 (1%)	-	0.10
Severe hypokalaemia - n (%)	4 (4%)	5 (4%)	9 (4%)	1.12 (0.29, 4.31)	0.87
Change in sodium at day 7 visit	10.9 (7.3)	11.3 (8.0)	11.1 (7.7)	-0.0 (-1.4, 1.4)	0.99
Change in potassium ((mmol/l) at day 7 visit	1.6 (1.2)	1.5 (1.2)	1.5 (1.2)	-0.0 (-0.3, 0.2)	0.73
Time to correction of severe hyponatraemia$^{\alpha}$				1.16 (0.88, 1.53)	0.30
Time to correction of severe hypokalaemia$^{\alpha}$				1.19 (0.86, 1.66)	0.30
**Anthropometry (secondary outcomes)**					
**Day 3**					
Change in weight (kg) - mean (sd) ‡	0.4 (0.2)	0.4 (0.3)	0.4 (0.2)	0.0 (-0.0, 0.1)	0.32
Change in weight-for-age z-score - mean (sd) ‡	0.5 (0.3)	0.5 (0.3)	0.5 (0.3)	0.0 (-0.0, 0.1)	0.22
Change in MUAC (cm) - mean (sd) ‡	0.2 (0.4)	0.3 (0.4)	0.3 (0.4)	0.1 (-0.0, 0.1)	0.17
Change in MUAC-for-age z-score - mean (sd) ‡	0.3 (0.4)	0.3 (0.4)	0.3 (0.4)	0.1 (-0.0, 0.2)	0.18
**Day 7**					

	ReSoMal (N=137)	WHO-ORS (N=135)	Overall (N=272)	Estimate of effect* (Standard ORS vs ReSoMal) (95% CI)	p value
Change in weight (kg) - mean (sd)	0.6 (0.4)	0.6 (0.4)	0.6 (0.4)	0.0 (-0.0, 0.1)	0.31
Change in weight-for-age z-score - mean (sd)	0.7 (0.4)	0.8 (0.4)	0.7 (0.4)	0.1 (-0.0, 0.2)	0.16
Change in MUAC (cm) - mean (sd)	0.6 (0.5)	0.6 (0.6)	0.6 (0.6)	0.1 (-0.0, 0.2)	0.21
Change in MUAC-for-age z-score - mean (sd)	0.6 (0.6)	0.7 (0.6)	0.6 (0.6)	0.1 (-0.0, 0.2)	0.18

*Continuous outcomes summarised with mean (sd) and estimate of effect analysed with linear regression adjusted for site, strata, and baseline values. Binary outcomes are summarised by N (%) and estimate of effects are unadjusted Mantel Haenszel odds ratios. Where there are 0 in a group, p-values are from Fisher's exact test.
[†]Hazard ratio from Cox model adjusted for site and strata.
[α]Time to correction of hyponatraemia and hypokalaemia were calculated using competing risks regression taking into account death, adjusted for site and strata.
[‡]Outcome not pre-specified in SAP.

Tables S8 Outcomes Stratum B: Moderate dehydration

	ReSoMal (N=69)	WHO-ORS (N=74)	Overall (N=143)	Estimate of effect* (Standard ORS vs ReSoMal) (95% CI)	p value
**Primary outcome (N with data)**	66	68	134		
Change in sodium at 24 hours	2.2 (7.2)	3.6 (8.4)	2.9 (7.8)	0.9 (-1.4, 3.2)	0.43
**Secondary outcomes**					
Mortality at 28 days	6 (9%)	6 (8%)	12 (8%)	0.85 (0.27, 2.63)†	0.77
Suspected pulmonary oedema - n (%)	0 (0%)	0 (0%)	0 (0%)		
Secondary heart failure - n (%)	0 (0%)	0 (0%)	0 (0%)		
**Biochemistry**					
**8 hours**					
Severe hyponatraemia - n (%)	15 (22%)	12 (18%)	27 (20%)	0.74 (0.32, 1.74)	0.49
Hypernatraemia - n (%)	3 (4%)	0 (0%)	3 (2%)	-	0.12
Severe hypokalaemia - n (%)	13 (19%)	18 (26%)	31 (23%)	1.50 (0.66, 3.38)	0.33
Change in sodium at 8 hours	0.7 (6.3)	0.9 (5.2)	0.8 (5.7)	0.2 (-1.6, 2.0)	0.87
Change in potassium at 8 hours	0.4 (0.9)	0.1 (0.9)	0.3 (0.9)	-0.3 (-0.6, -0.1)	0.013
**24 hours**					
Severe hyponatraemia - n (%)	12 (18%)	11 (16%)	23 (17%)	0.85 (0.35, 2.10)	0.73
Hypernatraemia - n (%)	2 (3%)	3 (4%)	5 (4%)	1.45 (0.23, 9.07)	0.69
Severe hypokalaemia - n (%)	5 (8%)	15 (22%)	20 (15%)	3.39 (1.13, 10.21)	0.028
Change in potassium at 24 hours	0.7 (0.7)	0.1 (1.0)	0.4 (0.9)	-0.5 (-0.8, -0.3)	0.0001
**7 days**					
Severe hyponatraemia - n (%)	1 (2%)	3 (8%)	4 (5%)	3.62 (0.35, 37.67)	0.25
Hypernatraemia - n (%)	1 (2%)	0 (0%)	1 (1%)	-	1.00
Severe hypokalaemia - n (%)	0 (0%)	1 (3%)	1 (1%)	-	0.49
Change in sodium at day 7 visit	5.7 (8.7)	6.7 (8.3)	6.2 (8.5)	-0.6 (-3.1, 1.8)	0.60
Change in potassium at day 7 visit	1.4 (1.2)	1.3 (1.3)	1.3 (1.2)	-0.2 (-0.6, 0.2)	0.33
Time to correction of severe hyponatraemia $^{\alpha}$				1.01 (0.59, 1.73)	0.97
Time to correction of severe hypokalaemia $^{\alpha}$				0.79 (0.47, 1.33)	0.38
**Anthropometry (secondary outcomes)**					
**Day 3**					
Change in weight (kg) - mean (sd)	0.3 (0.3)	0.3 (0.3)	0.3 (0.3)	0.0 (-0.1, 0.1)	0.35
Change in weight-for-age z-score - mean (sd)	0.4 (0.4)	0.4 (0.3)	0.4 (0.4)	0.0 (-0.1, 0.1)	0.65

	ReSoMal (N=69)	WHO-ORS (N=74)	Overall (N=143)	Estimate of effect* (Standard ORS vs ReSoMal) (95% CI)	p value
Change in MUAC (cm) - mean (sd)	0.3 (0.4)	0.1 (0.4)	0.2 (0.4)	-0.1 (-0.3, -0.0)	0.029
Change in MUAC-for-age z-score - mean (sd)	0.3 (0.4)	0.1 (0.4)	0.2 (0.4)	-0.2 (-0.3, -0.0)	0.012
**Day 7**					
Change in weight (kg) - mean (sd)	0.6 (0.6)	0.5 (0.5)	0.6 (0.5)	-0.1 (-0.2, 0.1)	0.41
Change in weight-for-age z-score - mean (sd)	0.7 (0.6)	0.6 (0.5)	0.7 (0.5)	-0.1 (-0.2, 0.1)	0.56
Change in MUAC (cm) - mean (sd)	0.6 (0.8)	0.4 (0.6)	0.5 (0.7)	-0.1 (-0.3, 0.1)	0.15
Change in MUAC-for-age z-score - mean (sd)	0.6 (0.8)	0.5 (0.7)	0.6 (0.8)	-0.1 (-0.4, 0.1)	0.21

*Continuous outcomes summarised with mean (sd) and estimate of effect analysed with linear regression adjusted for site, strata, and baseline values. Binary outcomes are summarised by N (%) and estimate of effects are unadjusted Mantel Haenszel odds ratios. Where there are 0 in a group, p-values are from Fisher's exact test.
[†]Hazard ratio from Cox model adjusted for site and strata.
[α]Time to correction of hyponatraemia and hypokalaemia were calculated using competing risks regression taking into account death, adjusted for site and strata.
[‡]Outcome not pre-specified in SAP.

Table S9 Post-Hoc Analysis: mortality at day 3 and day 7

	ReSoMal (N=206)	WHO-ORS (N=209)	Overall	Risk ratio (ORS vs ReSoMal) (95% CI)	p value
**Strata A + B**					
Mortality at day 3	14 (7%)	9 (4%)	23 (6%)	0.63 (0.28, 1.43)	0.28
Mortality at day 7	18 (9%)	17 (8%)	35 (8%)	0.93 (0.49, 1.76)	0.82
**Strata A only**					
Mortality at day 3	9 (7%)	7 (5%)	16 (6%)	0.79 (0.30, 2.06)	0.63
Mortality at day 7	12 (9%)	12 (9%)	24 (9%)	1.01 (0.47, 2.18)	0.97
**Strata B only**					
Mortality at day 3	5 (7%)	2 (3%)	7 (5%)	0.37 (0.07, 1.86)	0.21
Mortality at day 7	6 (9%)	5 (7%)	11 (8%)	0.78 (0.25, 2.43)	0.66

Table S10. Pre-specified sub-group analyses for the primary outcome of change in sodium at 24 hours

Subgroup		ReSoMal mean (sd)	WHO-ORS mean (sd)	Difference between arms mean (95% CI)	Interaction p-value
IV randomisation	WHO SAM	4.8 (7.9)	4.1 (6.9)	-0.7 (-2.8, 1.5)	0.39
	Plan C and Slow	8.9 (7.7)	7.4 (6.7)	-2.0 (-4.2, 0.1)	
Age	<1	7.2 (8.2)	5.0 (8.0)	-2.3 (-4.4, -0.3)	0.038
	≥1	4.1 (7.8)	5.0 (7.3)	0.5 (-1.2, 2.2)	
Consciousness level	A	4.5 (7.5)	4.8 (7.4)	-0.1 (-1.6, 1.4)	0.17
	V	7.6 (7.2)	6.9 (7.9)	-1.5 (-4.5, 1.5)	
	P/U	8.3 (16.3)	0.9 (7.5)	-5.7 (-11.9, 0.4)	
Respiratory distress	No	5.0 (7.9)	5.1 (7.4)	-0.4 (-1.8, 1.0)	0.41
	Yes	7.4 (8.9)	5.0 (8.7)	-2.1 (-5.8, 1.7)	
HIV positive	No	5.3 (8.1)	5.1 (7.6)	-0.6 (-1.9, 0.8)	0.89
	Yes	3.5 (9.9)	3.1 (4.2)	-1.2 (-9.6, 7.3)	
Strata	A	6.9 (8.0)	5.8 (7.0)	-1.3 (-3.0, 0.3)	0.13
	B	2.2 (7.2)	3.6 (8.4)	0.8 (-1.4, 3.1)	

Figure S1 Trial Flow

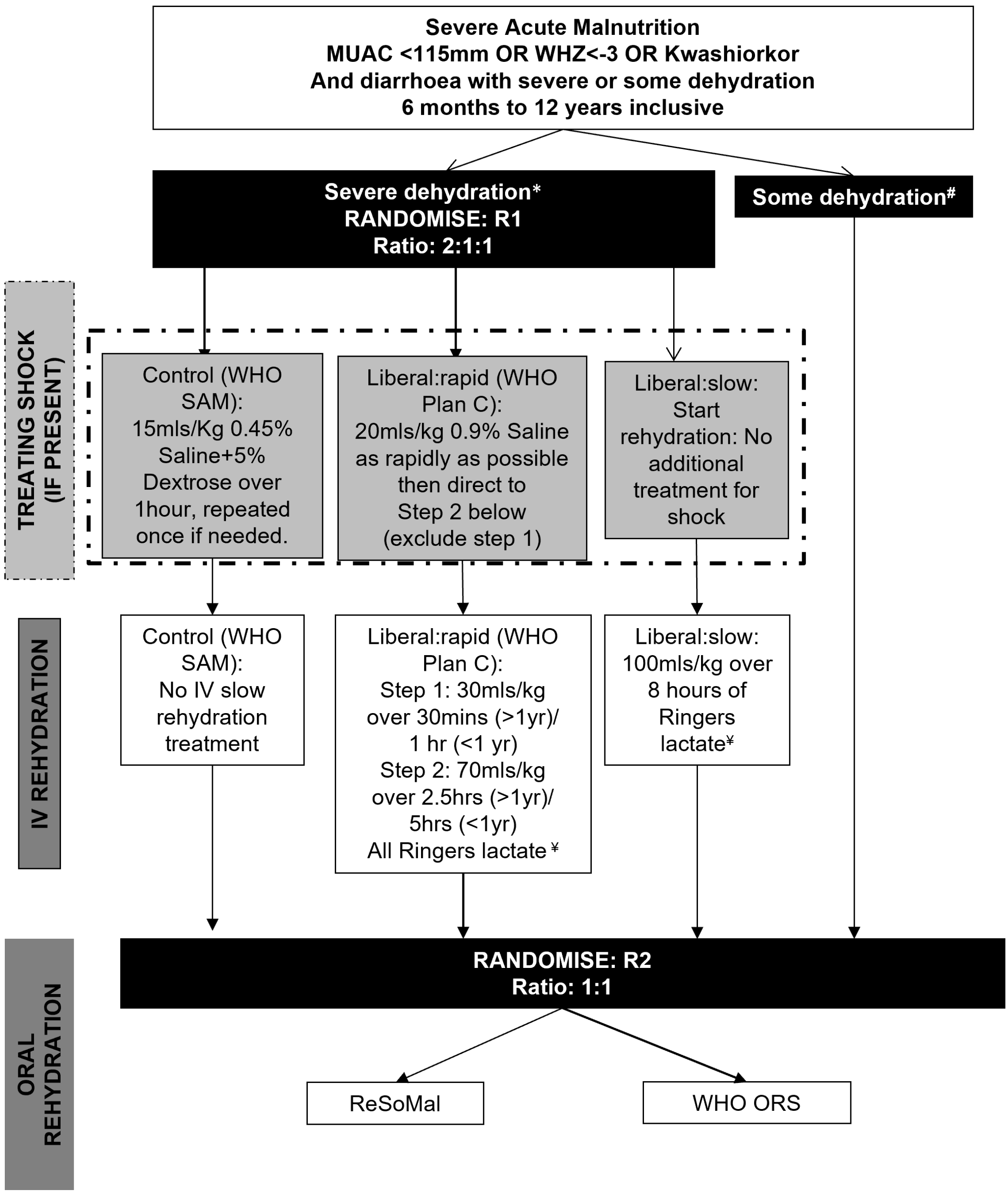

Assessment of severity of dehydration as per WHO 2013 Pocketbook
*All children receiving IV fluids for severe dehydration (R1) will also be randomised as per oral rehydration (R2).
#All children who present with 'some' dehydration will be randomised as per R2. If they go on to develop severe dehydration during admission, they will follow current WHO SAM guidelines.
¥Ringer's lactate/5% Dextrose can be used in place of Ringer's lactate

References

1. Maitland K, Kiguli S, Opoka RO, et al. Mortality after fluid bolus in African children with severe infection. N Engl J Med 2011;364(26):2483-95. DOI: 10.1056/NEJMoa1101549.
2. Talbert A, Thuo N, Karisa J, et al. Diarrhoea complicating severe acute malnutrition in Kenyan children: a prospective descriptive study of risk factors and outcome. PLoS One 2012;7(6):e38321. DOI: 10.1371/journal.pone.0038321.
3. Alam NH, Hamadani JD, Dewan N, Fuchs GJ. Efficacy and safety of a modified oral rehydration solution (ReSoMaL) in the treatment of severely malnourished children with watery diarrhea. J Pediatr 2003;143(5):614-9. DOI: 10.1067/S0022-3476(03)00500-6.
4. Kumar R, Kumar P, Aneja S, Kumar V, Rehan HS. Safety and Efficacy of Low-osmolarity ORS vs. Modified Rehydration Solution for Malnourished Children for Treatment of Children with Severe Acute Malnutrition and Diarrhea: A Randomized Controlled Trial. J Trop Pediatr 2015;61(6):435-41. DOI: 10.1093/tropej/fmv054.

Trial Protocol

Trial Statistical Analysis Plan
