# Supplementary material for: Oral rehydration for severe malnutrition in children with moderate and severe dehydration (GASTROSAM): a phase 2, open-label, superiority randomised controlled trial
Source: Lancet Child Adolesc Health. Author manuscript; Available in PMC 2026 Sep 29. (PMC7619504; doi:10.1016/S2352-4642(25)00371-2)
Supplement: Supplementary file 2 [file EMS216163-supplement-S1.pdf]

THE LANCET
Child & Adolescent Health

Supplementary appendix 2

This Equitable Partnership Declaration (EPD) was submitted by the authors, and we reproduce it as supplied. It has not been peer reviewed. *The Lancet*'s editorial processes have not been applied to the EPD.

Supplement to: Maitland K, Sainna H, Connon R, et al. Oral rehydration for severe malnutrition in children with moderate and severe dehydration (GASTROSAM): a phase 2, open-label, superiority randomised controlled trial. *Lancet Child Adolesc Health* 2026; published online March 10. https://doi.org/10.1016/S2352-4642(25)00371-2.

<u>GASTROSAM: Equitable Partnership Declaration</u>.

<u>Researcher considerations</u>

1. Please detail the involvement that researchers who are based in the country or countries of study had during a) study design; b) clinical study processes, such as processing blood samples, prescribing medication, or patient recruitment; c) data interpretation; and d) manuscript preparation, commenting on all aspects. If they were not involved in any of these aspects, please explain why.

**a) Study design:**<br>MH (an investigator who is Zambian and is based in Kenya), POO and FA (both born and working in Uganda) were involved in the design and operationalisation of the original Phase II clinical trial. The protocol was amended following consultation with Médecins Sans Frontières (MSF) involving AC and OFO (who are Nigerian working in Nigeria and MSF operational headquarters respectively) and SO (born in Burkino Faso and working in Niger). The protocol was approved by all members of the Trial Steering Committee (TSC): including IL (born and working in Uganda) and WM (born and working in Kenya). The trial, including the protocol, was designed to directly address one of the key knowledge gaps in management of severe malnutrition and was fully supported by the hospital sites, regional and national stakeholders in Kenya, Uganda, Niger and Nigeria.
**b) Clinical study processes:**<br>The clinical trial was exclusively conducted in four countries in Africa (Kenya, Uganda, Niger and Nigeria). SO and HS (Nigeria); SO and ASM (Niger); WO and DA (Uganda), EO, EM (Kenya) gathered the data; OFO ,POO, FA and MH supervised the respective study teams. The clinical trial management was conducted by the Kilifi Clinical Trials Facility (KCTF), Kenya (overseen by EO: born and working in Kenya), MH and KM (born in the United Kingdon working in Kenya). The data were entered into the RedCap database by local data entry teams (Uganda and Kenya) and the KCTF data entry teams entered data from Nigeria and Niger. The database was written by CM (born and working in Kenya) who oversaw the data management of the trial. Laboratory samples were processed in the sites by local study staff. Serious adverse event reviewing was overseen by EO (Kenya). Trial Steering Committee (TSC) included IL (born and working in Uganda) and WM (born and working in Kenya) who over saw trial governance. The data monitoring committee (DMC) included two members EM and PM who were born and work in Uganda.
**c) Data interpretation:**<br>Data analysis was supported by CM. Data interpretation was primarily conducted by RC, ECG (UK) and KM. The final data were presented at the 2-day results meeting hosted in Kenya by RC, CM, EO, ECG and KM. All listed investigators attended the results meeting in person or virtually, and had a chance to discuss the results and interpretation. A small briefing paper, summarising the findings, was prepared at this meeting, which all authors reviewed and approved. The briefing paper was shared with the hospitals which were involved in the trial and local Ministries of Health (Nigeria and Niger). The final results (and the final manuscript) were also presented separately to the TSC and DMC for feedback and approval.
**d) Manuscript preparation:**<br>GASTROSAM had a manuscript writing group which included KM, HS, SO, TS, OFO, ASM, CM, JED, IC, DMG, MEC, RC and ECG. The first draft was prepared by KM who shared it with the manuscript writing group. An advanced draft was shared with all investigators for feedback and comments.

2. How was funding used to remunerate and enhance the skills of researchers in the countries of study? And how was funding used to improve research infrastructure at the study sites?

**Skills:** The trial was run in four sites in Uganda (n=2) and Kenya n=2) who senior investigators (POO, FA in Uganda) and (EO and MH in Kenya) have a long track record of conducting GCP-compliant clinical trials. The bedside study teams recruited to conduct the trial at these sites were research naïve so completed online GCP and ethics courses (with certification) as well as trained for the trial on emergency triage and treatment (ETAT courses); clinical sign recognition (specific online training); dedicated ethics course for obtaining consent and research methods course for clinical trials including reporting procedures for serious adverse events. The sites in Niger and Niger were both research naïve so they required intensive training in trial conduct as well as completing the above courses. Opportunities for future employment have been realised by study staff owing to their training and experiences acquired during the conduct of the trial (references provided by supervisors).
**Research infrastructure:**<br>In the Médecins Sans Frontières units in Niger and Nigeria high dependency wards were strengthened and additional staff recruited to ensure rigorous and ethical trial conduct. Study specific equipment (such as bedside monitors, point of care diagnostics and anthropometry equipment) were purchased for the trial and donated to the hospital at the end of the study.

3. How did you safeguard the researchers who implemented the study?

The institutions involved in the trial have in place appropriate due diligence procedures which include safe-guarding, bullying and whistle blowing.<br>Workers employed on the trial worked on a shift-basis which was locally managed in accordance with institutional policies. PPE was available if required.

Benefits to the communities and regions of study

4. How does the study address the research and policy priorities of its location?

This is a key research gap that have been identified by the World Health Organization and Médecins Sans Frontières. The GASTROSAM trial addressed simultaneously two aspects of rehydration management (intravenous and oral rehydration) in a factorial trial. The findings are important since the current recommendations for management of dehydration are not based on evidence from clinical trials. These are likely to inform the current reviews of both of these aspects of management in the locations where the trial was run and in the region as a whole.

5. How will research products be shared in the community of study?

An aspect of knowledge sharing is **ensuring the results are available in formats that are accessible and appropriate to all key audiences**. For scientific audience we will publish in open access journals and provide access to sharing data by curating and depositing data from GASTROSAM in the Infectious Disease Data Observatory (IDDO) or KEMRI-Wellcome Dataverse data repositories.<br>For policy makers and health workers we plan to present results in other formats, such as policy briefs aimed at national policymakers that provide a clear interpretation of the results to feed into training programmes and guidelines for child health practitioners. These will be translated into French and Portuguese. We will commission a short video to communicate the results of that trial (see Picturing Health for other examples of our research: TRACT, FEAST trials), and plan to hold a webinar for communicating the results to relevant stakeholders communities and health professionals.<br>For local communities and patient representatives, we will provide lay summaries and feed these through meetings with the local communities involving hospital community representatives involved in the Community Advisory Board (CAB).

6. How were individuals, communities, and environments protected from harm?

a) *How did you ensure that sensitive patient data were handled safely and respectfully? Was there any potential for stigma or discrimination against participants arising from any of the procedures or outcomes of the study?*

All clinical data is held confidentially. Participants' unique identification number was collected as part of the trial initial and follow-up procedures. This was explained within the patient information sheet to reassure that the data are confidential and thus protect against stigmatisation.<br>The Kilifi Clinical Trial Facility, Kenya and MRC CTU at UCL, who oversaw the data management preserved the confidentiality of participants involved, which complied with requirements for data protection in the countries where the research is being conducted. All data was anonymised prior to presentation or publication of any results.

b) *Might any of the tests be experienced as invasive or culturally insensitive?*

We were required to place in intravenous canulae as part of the standard management. The initial (baseline) blood draw was taken during the process of cannula insertion. Minimal additional blood taking after this was required by the protocol (end points for clinical chemistry). All volumes of blood taken for research were below the maximum level recommended by local ethics committees. The patient information sheet had detailed explanation about why extra blood was required and an option was given to participants or their legal guardians to refuse additional research bloods.

c) *How did you determine that work was sensitive to traditions, restrictions, and considerations of all cultural and religious groups in the study population?*

The trial had broad eligibility criteria which were appropriate to the condition being studied. We did not collect data on ethnic origin or religion, only country of origin.

d) *Were biowaste and radioactive waste disposed of in accordance with local laws?*

Yes, biological samples were destroyed in accordance with institutional polices which are legally compliant. No radioactive waste was generated by the study.

e) *Were any structures built that would have impacted members of the community or the environment (such as handwashing facilities in a public space)? If so, how did you ensure that you had appropriate community buy-in?*

Not applicable. The study was conducted within hospitals.

f) *How might the study have impacted existing health-care resources (such as staff workloads, use of equipment that is typically employed elsewhere, or reallocation of public funds)?*

All study activity including personnel was funded directly. No public funds were used to support the trial.

7. Confirm that local ethics review was sought, and please provide the approval number. If not sought, please explain why.

The following ethic committees approved the trial<br>Imperial College Research Ethics Committee (Trial Sponsor)<br>Medical School Building<br>St Marys Campus London<br>W2 1PG UK<br>Protocol Number: 18IC4427<br>Date 10/04/2018<br><br>Mbale Regional Hospital Research and Ethics Committee

Pallisa Road
Mbale PO Box 921
Uganda
Protocol Number MRRH-REC OUT 107/2018
Date 14/06/2019

KEMRI Scientific and Ethics Committee
Mbagathi Road
POB584840
Nairobi
Kenya
Protocol Number: KEMR/SERU/CGMR-C/204/4072
Date 25/08/2020

Médecins Sans Frontières (MSF) Ethics Committee*
Arbre Benit Street
Brussels
1050
Belgium
Protocol Number: ERB 2293
Date 25/07/2023

Comité National pour l'Ethique en Recherche de Santé
Ministere De la Sante Publique
Rupublique de Niger
Niamy
Protocol Number 47/2023/CNERS
Date 08/08/20023

Nigeria NHREC (National Health Research Ethics Committee)
Ministry of Health
Abuja
NHREC Protocol Number NHREC/01/01/2007- 22/09/2023
NHREC Approval Number NHREC/01/01/2007
Date 04/10/2023

Secondary analyses

8. Have the data analysed in your study been extracted from another source, such as a national survey, rather than being directly collected by the authors of this paper?

N/A

If the authors of this paper were not involved in data collection, how were the findings interpreted with sufficient contextual knowledge?
The Lancet Child & Adolescent Health *believe contextual understanding is crucial for informed data analysis and interpretation.*

N/A

9. Finally, please provide the title (eg, Dr/Prof, Mr/Mrs/Ms/Mx), name, and email address of an author who can be contacted about this statement.

Name: Professor Kathryn Maitland
Email: k.maitland@imperial.ac.uk
